# The role of the gut microbiota in health and cardiovascular diseases

**DOI:** 10.1186/s43556-022-00091-2

**Published:** 2022-10-11

**Authors:** Lu Wang, Shiqi Wang, Qing Zhang, Chengqi He, Chenying Fu, Quan Wei

**Affiliations:** 1 Rehabilitation Medicine Center and Institute of Rehabilitation Medicine, West China Hospital, Sichuan University, Chengdu, People’s Republic of China; 2 Key Laboratory of Rehabilitation Medicine in Sichuan Province, Chengdu, People’s Republic of China; 3 National Clinical Research Center for Geriatrics, West China Hospital, Sichuan University, Chengdu, People’s Republic of China; 4 Aging and Geriatric Mechanism Laboratory, West China Hospital, Sichuan University, Chengdu, People’s Republic of China

**Keywords:** Gut microbiota, Dysbiosis, Metabolites, Cardiovascular Diseases, Risk factors, Health

## Abstract

The gut microbiota is critical to human health, such as digesting nutrients, forming the intestinal epithelial barrier, regulating immune function, producing vitamins and hormones, and producing metabolites to interact with the host. Meanwhile, increasing evidence indicates that the gut microbiota has a strong correlation with the occurrence, progression and treatment of cardiovascular diseases (CVDs). In patients with CVDs and corresponding risk factors, the composition and ratio of gut microbiota have significant differences compared with their healthy counterparts. Therefore, gut microbiota dysbiosis, gut microbiota-generated metabolites, and the related signaling pathway may serve as explanations for some of the mechanisms about the occurrence and development of CVDs. Several studies have also demonstrated that many traditional and latest therapeutic treatments of CVDs are associated with the gut microbiota and its generated metabolites and related signaling pathways. Given that information, we summarized the latest advances in the current research regarding the effect of gut microbiota on health, the main cardiovascular risk factors, and CVDs, highlighted the roles and mechanisms of several metabolites, and introduced corresponding promising treatments for CVDs regarding the gut microbiota. Therefore, this review mainly focuses on exploring the role of gut microbiota related metabolites and their therapeutic potential in CVDs, which may eventually provide better solutions in the development of therapeutic treatment as well as the prevention of CVDs.

## Introduction

Trillions of microorganisms colonize the anaerobic and nutritious environment in the gut to form a healthy intestinal physiological ecosystem [1]. These communities are regarded as “gut microbiota”, and the sum total of all microorganism genomes in the gut, including their DNA sequences and other genetic information, are together called “gut microbiomes” [2]. Most intestinal microbial communities in the human intestinal tract are bacteria with complex structures [3]. There are more than 1,000 species of intestinal bacteria, and the number reaches approximately 10^14^. The ratio of bacterial number to human cell number ranged from 10:1 to 1:1 [4]. It also contains 100-fold more genes than our own genome [5], which is called the second genome of the human body [6]. In terms of the types of intestinal bacteria, the gut microbiota is mainly composed of *Firmicutes*, *Bacteroidetes, Proteobacteria* and *Actinobacteria*, and this composition is relatively stable in healthy individuals but not in patients with cardiovascular diseases (CVDs) or other diseases [2, 7, 8]. Among those gut microbiota, *Firmicutes* and *Bacteroidetes* in the large intestine account for about 90% of the total number of gut microbiota, and the ratio is a vital health indicator reflecting the condition of health and is also associated with the incidence of CVDs [9]. In addition, the gut microbiota maintains a symbiotic or antagonistic relationship with its host to form a dynamic and balanced microbial system.

To date, establishing a clear and direct relationship between gut microbiota and corresponding diseases may be challenging due to the mixture of other non-host genes from viruses, fungi, and archaea [10]. Fortunately, advanced sequencing technologies including 16S rRNA and metagenome, have been used with blood or fecal samples to determine pathogenic non-host genes [11]. More importantly, the diversity and metabolites of normal gut microbiota are closely related to human health, and the imbalanced gut microbiota plays an important role in the occurrence and development of human diseases, in which the impact of gut microbiota on CVDs receives increasing attention [2].

In this review, we summarized the latest literature in order to explore the role of gut microbiota in physical conditions, pathological dysbiosis, and metabolites participating in the occurrence, development, and the treatment of CVDs. Herein, we systematically described the influence of gut microbiota on health, including its main function and material metabolism, and then discussed the influence of major metabolites produced by gut microbiota on several common cardiovascular risk factors and the main CVDs in depth, thereby gaining a comprehensive understanding of the pathogenesis and mechanism of CVDs. Eventually, we also reviewed and elaborated evidence about promising methods such as diet intervention, for the prevention and treatment of CVDs, and more importantly, targeting gut microbiota and its metabolites will be a novel method for the prevention and treatment of CVDs.

## The role of the gut microbiota in health and diseases

Studies have shown that the fetus was exposed to bacteria before birth [12] and the anaerobic bacteria colonize the fetus during pregnancy [13]. The traditional concept is that the uterus is a sterile environment, and after the fetus is born, the intestinal microbiota is gradually colonized due to breast milk and other food feeding [14]. As time goes on, the abundance and diversity of gut microbiota increased in the neonatal period [15], and in early childhood, the diversity gradually formed and stabilized [16]. Consequently, in healthy individuals, these microbes compete and restrict each other to maintain a normal dynamic equilibrium state [17].

The bacteria living in the human gut are also called commensals. Some beneficial commensal microbes could repair the normal function of the intestinal barrier and exert anti-inflammatory effects, such as *Akkermansia muciniphila (A. muciniphila)*, *Faecalibacterium prausnitzii,* and *Roseburia intestinalis,* et al. [18]*.* These commensal microbes make up about 20% of the total gut microbiota. They are indispensable for maintaining the physiological function of adult tissues and organs by synthesizing a variety of vitamins, participating in food digestion, producing lactic acid, promoting intestinal peristalsis, inhibiting the growth of pathogenic microbiota, and activating the immune system [19]. For example, one of the most important commensal microbes in the human gut is *Bifidobacterium*, a strictly anaerobic gram-positive bacterium [19], which assists the human body in the digestion and absorption of nutrients, resists the invasion of harmful bacteria, improves the immune function of the body, secretes molecules to regulate immune function, and participates in the metabolism of substances in the intestine [20, 21]. Besides, another common commensal microbe, *A. muciniphila*, a gram-positive bacterium, reduces insulin sensitivity and is inversely associated with the development of obesity and diabetes [22]. Furthermore, *Clostridium butyricum* can produce butyric acid, improve insulin sensitivity, produce satiety, and reduce the content of adipose tissue [23, 24].

Additionally, other commensal bacteria such as *Escherichia coli*, *Lactobacillus*, *Streptococcus, Helicobacter pylori, segmented filamentous Bacteria,* and enterotoxigenic B. *fragilis* are called pathobionts [25]. They make up about 70% of the total gut microbiota and interact with the host to regulate the immune response [25]. They are harmless under physical conditions [26], and have a potential pathogenic influence on the host following changes in the environment [27]. However, in the case of immune dysfunction and other pathological conditions, these pathobionts can greatly proliferate in a short time period, or translocate from the intestine to other parts of the body to be pathogenic [28].

Given that the gut microbiota is essential in human health, changes in the composition and function of the gut microbiota may result in the development of diseases [29]. Due to the influence of host genes, diet, antibiotic use, lifestyle, drugs as well as other factors [30], the composition and diversity of gut microbiota gradually change with interindividual variation [30, 31]. For example, a high-fat diet leads to an increase in the *Firmicutes* to *Bacteroidetes* ratio [32]. When the composition and proportion of gut microbiota change, inflammation and metabolic abnormalities can be induced, which contribute to the development of different diseases [33, 34]. Dysbiosis of gut microbiota refers to changes in the number, species and composition of gut microbiota [35]. In recent years, gut microbiota dysbiosis has been shown to be critically involved in health and has emerged as a potential pathogenesis for various diseases, such as inflammatory bowel disease, cancers, and CVDs [36, 37]. Moreover, gut dysbiosis is also a driver of metabolic inflammation and metabolic dysregulation, which serve as a key feature of metabolic diseases [38]. For instance, the translocation of intestinal bacteria to the blood and liver through the portal venous system together with its metabolites are able to stimulate the release of inflammatory factors, and promote the occurrence of non-alcoholic fatty liver as well as hepatitis [39]. Moreover, it has been reported that the gut microbiota also plays an important role in CVDs [40, 41], which has aroused our great interest.

Herein, we introduce the main function of gut microbiota interacting with the host by regulating the intestinal mucosa barrier and immune homeostasis, and metabolism of nutrients (glucose, lipids, and protein) to explore the role of gut microbiota in health. Then, we provide information on how the gut microbiota promotes the development of diseases, especially CVDs.

### The gut microbiota regulates normal physiological functions

#### The gut microbiota maintains a normal intestinal mucosal barrier

Studies showed that the interaction between the gut microbiota and host is crucial for the host to maintain normal intestinal and physical function [42], involving the maintenance of intestinal barrier integrity, the growth and regulation of the immune system, and normal homeostasis [43, 44]. Commensal bacteria play an important role in regulating multiple physiological functions, including modulating the host’s gut mucosal barrier function, maintaining intact tight mucosal junctions, and regulating normal mucosal immunity [45]. One of the most important functions of the gut microbiota is establishing the normal intestinal mucosal barrier with other components in the host intestine [46]. The intestinal mucosal barrier is mainly composed of the intestinal epithelial cell junction complex and its secretions, immune cells, and gut microbiota [47]. The complete intestinal mucosal barrier can effectively block the colonization and invasion of pathobionts and maintain the stability of the intestinal environment [48, 49].

The gut microbiota in healthy people can influence intestinal epithelial cells to prevent the destruction of intestinal mucosa [50, 51]. It was found that butyrate derived from gut microbiota can regulate the repair of intestinal mucus barrier by activating the macrophage/wingless and int-1 (Wnt)/extracellular regulated protein kinase (ERK) signaling pathways which effectively separating the body from intestinal pathogens [52]. Other researchers further found that *Bifidobacterium brevis* could reduce dextran sodium sulfate-induced apoptosis of intestinal epithelial cells and reduce intestinal inflammation, which indicated that gut microbiota could protect intestinal mucosa by reducing apoptosis of intestinal epithelial cells [53]. Oral administration of commensal bacteria such as *Bifidobacterium* could also improve the function of intestinal barrier and inhibit the growth of pathobionts [54].

Furthermore, tight junctions are an important connection mode of intestinal epithelial cells and are considered to be a key component, which controls paracellular transport of semipermeable barriers in the small intestine and large intestine [55]. It was demonstrated that the expression level of intestinal epithelial tight junction protein regulatory factor zonulin was significantly increased in hypertensive patients with gut microbiota disorder [56]. Meanwhile, gut microbiota can also enhance the formation of tight junctions and regulate the permeability of intestinal epithelium [57, 58].

At present, it is generally believed that dysbiosis of the gut microbiota is an important factor affecting the integrity of the intestinal barrier [59]. Previous studies showed that the dysbiosis of the gut microbiota could lead to the rapid proliferation of intestinal pathogenic bacteria, and the pro-inflammatory factors released by them seriously damaged the structure and function of the intestinal barrier, and it also promoted the occurrence and development of diseases in the digestive system as well as metabolic system [60, 61]. Furthermore, the dysbiosis of the gut microbiota triggers intestinal mucosal barrier damage, leading to inflammation and disordered nutrient metabolism [62]. Overactivated inflammation caused by microbiota dysbiosis disrupts normal intestinal mucosal barrier function, and increases intestinal permeability to promote bacterial translocation, leading to endotoxemia and inflammation, thereby increasing the risk of CVDs [62].

#### The gut microbiota helps to maintain the immune homeostasis

The homeostasis between the gut microbiota and the host is a major modulator of the evolution in the mammalian immune system and the maturation of immunologic tissues [63]. In addition to barrier function, gut microbiota is closely related to health and diseases through regulating immune processes [64]. With advances in scientific and technological approaches for investigating the microbiota, study have revealed that the dynamic crosstalk between the host gut and microbiota is crucial for maintaining immune homeostasis [65]. The intestinal mucosal immune system is considered to be the largest immune component in the body, and is functionally related to the intestinal microbiota [66].

**Fig. 1:**
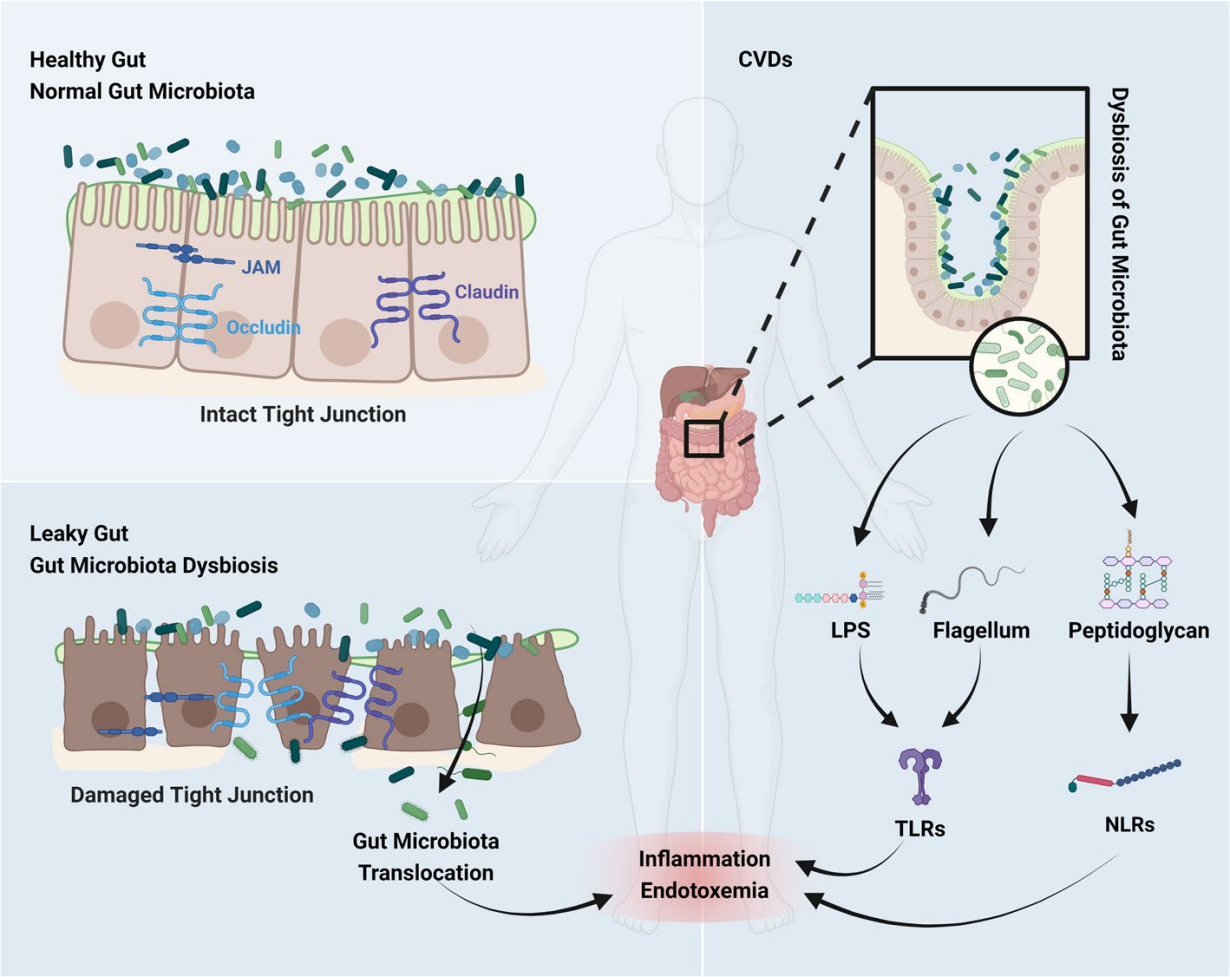
The gut microbiota helps maintaining the balance of the mucosal barrier and immune homeostasis (created with BioRender.com). In the healthy gut, the intact mucosa barrier and tight junction prevent the translocation of gut microbiota, while gut microbiota dysbiosis causes disease. Under the leaky gut caused by dysbiosis, the gut microbiota translocases into the blood; the components of microbiota such as LPS activate TLRs and NLRs induce inflammation, endotoxemia, and immune dysregulation, resulting the development of CVDs. Abbreviations: JAM, junctional adhesion molecules; LPS: lipopolysaccharide; TLRs: Toll like receptors; NLRs: Nod-like receptors

Recent evidence has demonstrated the importance of the gut microbiota in the formation and development of the immune system. The gut microbiota has profound effects on the formation of lymphoid tissue and the development of the immune system [67]. It stimulates the development of intestinal-associated lymphoid tissues, activates lymphocytes, and regulates the production of immunoglobulin A to defend against pathogens [27, 68]. The structural components of gut microbiota such as lipopolysaccharide (LPS), flagella, and peptidoglycan, can interact with receptors on human cells to stimulate and guide the host immune response [69]. For instance, LPS binds to Toll-like receptor (TLR) 4, flagella binds to TLR5, and peptidoglycan binds to TLR2 [70], triggering the expression of a large number of downstream inflammatory factors, thereby affecting the cardiovascular system [71]. Specially, LPS can interact with different receptors to induce inflammation and immune responses to increase intestinal permeability and CVDs susceptibility (Fig. 1).

Moreover, Nod-like receptors (NLRs) are also important pattern recognition receptors that are very important for the maintenance of the intestinal microecology and mucosal barrier [72]. Except for the TLR family, the role of the NLR family in CVDs susceptibility has now received increasing attention. The NLR family contains nucleotide-binding oligomerization domain 1 (NOD1), nucleotide-binding oligomerization domain 2 (NOD2) as well as NOD, leucine-rich repeat (LRR), and Pyrin domain-containing protein 3 (NLRP3) [73]. LPS released by gut microbiota can further promote the expression of NLRP3 [74]. In fact, after binding to its ligand, NLRs can induce the expression of cytokines and chemokines through the activation of nuclear factor kappa-B (NF-κB) pathway or mitogen-activated protein kinase (MAPK) pathway, and further induce the immune inflammatory response [75].

Importantly, studies found that the occurrence of CVDs was also involved in the immune response mediated by the NLR family [76, 77]. For example, atherosclerotic plaques in LDLR^−/−^ NOD 1/2^−/−^ mice showed less lipid deposition and macrophage aggregation, suggesting that lack of NOD 1/2 may reduce the occurrence of atherosclerosis [78]. Moreover, the peptidoglycan components in bacteria can bind to NOD1 and NOD2, which in turn mediate the occurrence of the inflammatory response, and further leads to atherosclerosis [77]. Besides, inflammation induced by the NLRP3 inflammasome plays a role in the interaction between gut microbiota and cardiometabolic diseases. For instance, the NLRP3 deficient obese mice had altered composition of the gut microbiota, decreased TMAO and LPS levels, as well as downregulated hepatic steatosis and myocardial energy metabolism [79]. Nevertheless, another study reported that NOD TLR4 ^−/−^ mice developed higher body weight, hyperlipidemia, severe insulin, and glucose intolerance, lower circulating SCFAs levels, higher levels of *Bacteroidetes*, lower levels of *Firmicutes* in the large intestine, as well as fewer SCFAs-associated gut microbiota, all of which may promote the development of insulin-deficient diabetes [80].

Given that the gut microbiota is important in the regulation of immune homeostasis, many studies have reported an association between the gut microbiota and the occurrence, and the treatment of immune-related diseases such as inflammation, cancers, and autoimmune diseases [81]. For example, among metastatic melanoma patients receiving immunotherapy, the diversity and composition of the gut microbiota differed significantly between responders and non-responders, and the function of the gut microbiota differed between the two groups [82]. Several interventions targeting gut microbiota, such as SCFAs supplementation and microbiota transplantation, have shown beneficial therapeutic efficacy for the treatment of inflammatory bowel disease [36, 83, 84]. Additionally, the gut microbiota also triggers immune dysregulation and chronic low-grade inflammation to promote the development of CVDs [85].

#### The gut microbiota modulates the neuroendocrine system

Additionally, it has been reported that the intestinal microbiota plays a very crucial role in the neuroendocrine system, which is correlated to the gut-brain axis [86, 87]. As the major neuroendocrine system, the hypothalamic–pituitary–adrenal (HPA) axis regulates various pathophysiological processes in response to inside and outside stressors. It is increasingly recognized that the establishment of intestinal microbiota in early life can impact the development as well as the function of nervous system, especially regulating neuroendocrine function [86, 87]. In neonatal germ-free mice, colonization with *B. infantis* [88], and *Bifidobacterium* [89] could attenuate the high sensitivity of the HPA axis [88] and establish functional neural circuits [89]. The imbalanced microbiota may fail to execute metabolic functions [90], brain function and neuromodulators [91]. The regulation of neurohumoral-immune not only promotes the development of neurological diseases, but also promotes the occurrence and development of CVDs via the HPA axis and renin-angiotensin system (RAS) [92]. For example, dysregulation of the gut-brain axis was associated with hypertension [93]. Interestingly, the gut microbiota can be called “an endocrine organ” that biologically regulates host metabolism [94], for affecting the host’s endocrine system by altering the functional metabolism of important hormones such as leptin, ghrelin and cortisol [95]. These hormones also have a great impact on human health and may cause diseases under abnormal circumstances.

In fact, changes in the function of gut microecology and gut microbiota may cause the occurrence and exacerbation of various diseases. It was found that dysbiosis of the gut microbiota may lead to decreased cardiac function and increased cardiomyopathy as well as cardiac insufficiency [2], which is of great significance in predicting CVDs and also has important predictive significance for the prognosis of adverse cardiovascular events. The gut microbiota has been reported to be associated with CVDs and its corresponding risk factors, such as obesity [96], diabetes mellitus [97], and insulin resistance [98]. These risk factors may affect the composition and the diversity of gut microbiota [99]. In addition, gut dysbiosis is also linked to inflammation, oxidative stress, platelet activity, thrombosis and atherosclerosis, which contribute to the progression of CVDs [100]. The possible mechanism of gut microbiota dysbiosis related to CVDs includes its role in increasing intestinal permeability and triggering inflammation via the LPS/TLR4 as well as NLRP3 pathways, thereby ultimately contributing to the development of CVDs [101]. In short, the inflammation directly triggers by gut microbiota is one of the possible mechanisms of CVDs susceptibility, and material metabolism affected by gut microbiota was proposed to play a role as well [102].

### The gut microbiota participates in material metabolism

In the human body, the gut microbiota can act on the metabolic system of the human body by participating in the metabolites of various nutrients [103–105]. The digestion and absorption of glucose, lipids, and amino acids by the intestinal tract supply the body with energy which in turn perform various life activities. The gut microbiota participates in food digestion and fermentation processes to regulate the energy harvesting process, and this helps to maintain the metabolic homeostasis and plays specific functions in host nutrient metabolism [106]. Moreover, it can utilize the provided metabolites to synthesize a variety of amino acids and vitamins to supplement required nutrients for the growth of the human body [107].

**Fig. 2:**
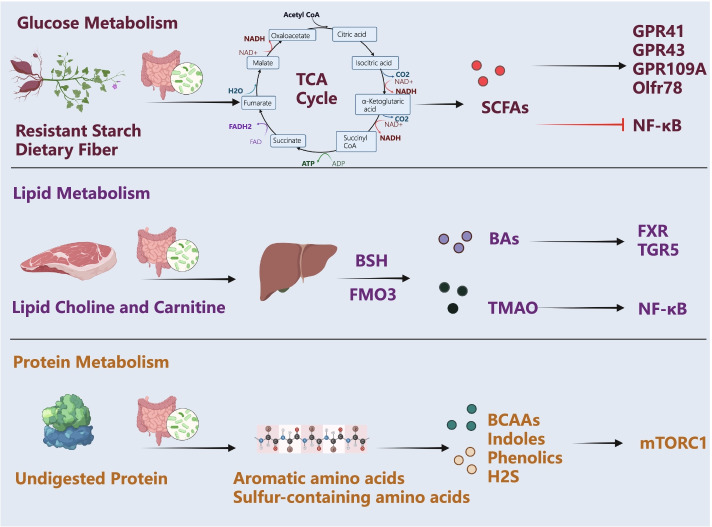
The gut microbiota participates in material metabolism to produce metabolites (created with BioRender.com). The gut microbiota can affect the metabolism of glucose, lipids, and proteins by generating a series of metabolites and activating downstream signaling pathways. Abbreviations: SCFAs: short-chain fatty acids; BAs, bile acids; TMAO: trimethylamine N-oxide; BCAAs: branched-chain amino acids; GPR, G protein-coupled receptor; Olfr78: olfactory receptor 78; NF-κB: nuclear factor kappa-B; BSH: bile salt hydrolase; FMO3: flavin monooxygenase 3; FXR: farnesoid X receptor; TGR5: Takeda-G-protein receptor 5; mTORC1: mammalian target of rapamycin complex 1

When the gut microbiota is dysregulated and the composition changes, the balance of glucose metabolism, lipid metabolism and protein metabolism are all destroyed, which leads to a series of changes in the production of corresponding metabolites (Fig. 2). These metabolites could maintain human health. Moreover, the concentration change in metabolites is associated with various diseases [108–110]. One of the crucial metabolites of glucose metabolism regulated by gut microbiota is short chain free acids (SCFAs) [111], which take part in the development of CVDs [112]. With the decreasing of SCFAs-generating gut microbiota, the homeostasis of glucose metabolism would be disrupted to promote the risk of obesity, diabetes, and other CVDs [113, 114]. Of note, other important metabolites of lipid metabolism generated by gut microbiota, bile acids (BAs) and trimethylamine-N-oxide (TMAO), are suggested to regulate the occurrence and development of CVDs [108–110]. Furthermore, undigested protein is first decomposed into amino acids under the action of intestinal bacteria [115]. There are many types of amino acid metabolites, but only a few of them have been studied thus far. Various amino acids can be deaminated and trans-aminated by intestinal bacteria to produce a variety of α-keto acids or α-hydroxy acids with different structures, and then undergo a series of complex redox reactions to produce a variety of products, mainly branched-chain amino acids (BCAAs), indoles, and phenolic compounds generated by aromatic amino acid metabolism and H_2_S generated by sulfur-containing amino acids [116, 117]. Excitedly, the metabolites of protein and amino acids are also associated with the development of CVDs. For example, the levels of BCAAs are associated with body composition and inflammatory factors [118]. Moreover, it has been reported that an imbalance in the ratio of BCAAs to tryptophan and threonine leads to increased appetite, and then increases the risk of obesity [119].

Herein, in this section, we take the metabolism of glucose, lipid, and amino acids as examples to hint at how gut microbiota produce metabolites in the process of material metabolism to modulate health and diseases, especially CVDs.

#### The gut microbiota regulates glucose metabolism through the production of SCFAs

Recently, many studies have reported that the gut microbiota has an impact on host nutrient metabolic health to trigger some diseases, especially the glucose metabolism [120]. In general, the gut microbiota was reported to regulate postprandial glucose after dietary intake [120]. However, the postprandial blood glucose level induced by different diets could also impact the composition of gut microbiota [121]. SCFAs is the main metabolite in the glucose metabolism and a high-fiber plant-based diet was reported to increase SCFAs-producing microbiota [122]. Besides, diet interventions, including a green-Mediterranean diet, and SCFAs supplementation may reshape the gut microbiota [123–125].

Mechanically, current studies suggest that SCFAs may exert their functions through acting as energy substrates and maintaining metabolic integration by a variety of different downstream regulatory mechanisms, such as G-protein-coupled receptors (GPRs) with GPR41/Recombinant Free Fatty Acid Receptor (FFAR3), GPR43/FFAR2, GPR109A, Olfactory Receptors 78 (Olfr78) in endocrine cells of the gut wall [126], as well as histone deacetylases (HDACs) [127, 128] (Fig. 2). GPR41 regulates host energy acquisition and promotes the catabolism of glucose and lipids [129]. The decreased SCFAs may cause less activated GPR41 and higher levels of energy uptake [129]. In addition, it has also been reported that the lower production of SCFAs in gut microbiota would increase the host’s capability of obtaining energy from the diet [130]. SCFAs from bacterial fermentation promote the secretion of glucagon-like peptide 1 (GLP-1) and peptide YY (PYY) through GPR43 and GPR41 from intestinal L cells [131–133]. GLP-1 increased pancreatic insulin secretion, and inhibited the production of glucagon. Deletion of GLP-1 abolished the beneficial effects of prebiotics on weight gain, glucose metabolism, and inflammatory pathway activation [134]. Moreover, PYY increases satiety, reduces food intake, inhibits intestinal motility, and increases the intestinal transit rate [129].

Particularly, SCFAs also have anti-inflammatory functions. It was reported that the butyrate and propionate can inhibit the tumor necrosis factor (TNF) and NF-κB signaling pathways [135]. These may be because that SCFAs can inhibit the activity of HDAC, inactivate the NF-κB pathway and decrease the expression of interleukin-2 (IL-2), IL-6, and TNF-α, which further control inflammation [113]. Meanwhile, butyrate increases the acetylation of H3 histone in the Forkhead box protein P3 (FOXP3) promoter by inhibiting HDAC4 [136], thus promoting Treg differentiation, affecting G1-phase-specific cyclins, and further resulting in the markedly inhibited proliferation of vascular smooth muscle cells, thereby inhibiting myocardial fibrosis and improving heart function [137].

#### The gut microbiota has an impact on lipid metabolites

It is well known that the balance of cholesterol is closely related to human health, and disorder of cholesterol balance leads to cardiovascular and metabolic diseases [138]. For example, low-density lipoprotein (LDL) cholesterol is ingested by macrophages to form foam cells and eventually leads to atherosclerosis, while high-density lipoprotein (HDL) can promote reverse cholesterol transport and play a cardiovascular protective role [139]. In recent years, gut microbiota is reported to be associated with levels of circulating triglycerides and HDL cholesterol [140].

Although the mechanisms by which the gut microbiota regulates lipid metabolism have not been fully clarified, BAs and TMAO have been well suggested to regulate lipid metabolism [108, 109]. Some gut microbiota can oxidize cholesterol in the intestine into cholestenone via cholesterol oxidase to accelerate cholesterol degradation [141, 142]. They can also participate in the metabolism of BAs, to achieve the indirect metabolism of cholesterol [143].

BAs, as by-products of cholesterol metabolism, are the main organic component of bile and are synthesized in hepatocytes [144]. Cholic acid (CA) and chenodeoxycholic acid (CDCA) are primary BAs directly synthesized from cholesterol; secondary BAs include deoxycholic acid (DCA), lithocholic acid (LCA), ursodeoxycholic acid (UDCA) and their glycine and taurine conjugated forms [145]. BAs are excreted into the intestine to aid in the digestion and absorption of lipids and combine with farnesoid X receptor (FXR) and takeda-G-protein receptor 5 (TGR5) [146] (Fig. 2). There is a strong biochemical relationship between BAs and gut microbiota. Some gut microbiota in healthy people can convert conjugated BAs into free BAs, and can transform BAs into secondary BAs with the help of bile salt hydrolase (BSH) and cholesterol 7-alpha hydroxy-lase (CYP7A1) [147], so as to reduce blood cholesterol by affecting the enterohepatic circulation of BAs [148, 149]. Besides, the gut microbiota affects the metabolism of BAs by regulating the activity of BSH to reduce LDL cholesterol levels [150]. BAs have bacteriostatic properties and an antimicrobial effect [151, 152]. BAs destroy the integrity of the bacterial cell membrane and thereby change the intestinal microecology [153]. BAs also prevent bacteria from overgrowth and decrease inflammation [154]. More importantly, BAs are also closely associated with CVDs [155]. For example, BAs levels in the feces of patients with CVDs are different from those in healthy individuals [156]. And BAs have been reported to regulate vascular tension and affect ion exchange on cardiomyocyte membranes, suggesting that regulating BAs by gut microbiota might be a treatment for CVDs [157, 158].

In addition, phosphatidylcholine can be hydrolyzed into choline in the intestine in vivo, and the gut microbiota can convert choline into trimethylamine (TMA) [94, 159]. After being absorbed by the intestinal tract, TMA is further transported to the liver and oxidized into TMAO by liver flavin monooxygenase 3 (FMO3), which plays an important role in human health and CVDs [94, 159]. TMAO also reduces reverse cholesterol transportation, and changes BAs composition [160]. Some studies genetically manipulated hepatic FMO3 to modulate lipid homeostasis [161], suggesting a major role of gut microbiota in lipid metabolism. Furthermore, changes in TMAO levels have been found in metabolic diseases such as fatty liver and hyperlipidemia, suggesting that lipid metabolism mediated by gut microbiota plays an important role in the occurrence and development of diseases [162, 163].

#### The correlation between gut microbiota and protein metabolism: the importance of small molecule metabolites

Under physical conditions, the gut microbiota maintains the balance of protein and amino acid metabolism [116, 117]. For example, fermentation of dietary polyphenols (mainly hydroxycinnamic acid and steroids) by gut microbiota (e.g., *Enterobacter, Bifidobacterium, Clostridium*) produces bioactive compounds composed of phenolic acids that can be absorbed by the gut [164].

BCAAs are essential amino acids that cannot be synthesized in the human body and are obtained from food, including leucine, isoleucine, and valine [165]. Red meat and dairy products are rich in BCAAs and synthesized by intestinal bacteria such as *Enterococcus, Enterobacter, Bis Fidobacterium, and Clostridium botulinum* [166]. Their structures are characterized by branched chains, which refer to one carbon in the center connected to three or more carbon atoms [167]. BCAAs are associated with insulin resistance, which is the main mechanism of the development of cardiometabolic disease [168] (Fig. 2).

Aromatic amino acids including tyrosine, tryptophan, and phenylalanine are metabolized into indole and phenols by certain intestinal anaerobic bacteria, such as *Bacteroidetes, Lactobacillus, Bifidobacterium, Clostridium,* and *Peptostreptococcus* [169]. Tryptophan is decomposed into indole, indoleacetic acid, indole-3-lactic acid, 3-methylindole (skatole) and other indole compounds under the action of tryptophanase. Tryptophanase is the key enzyme that generates indole [170]. The activity of the enzyme is easily induced by the substrate tryptophan, so when the amount of tryptophan changes in the colonic environment, the content of indole will fluctuate greatly [170]. Of note, the metabolic products of aromatic amino acids generated by gut microbiota have a great impact on the pathology of cardiometabolic diseases [171]. For example, indole propionic acid is associated with insulin sensitivity and appears to reduce the risk of diabetes [171]. Moreover, the gut microbiota metabolizes dietary tryptophan to indoxyl, which further generates indoxyl sulfate through sulfonation. Excessive accumulation of indoxyl sulfate causes cardiomyocyte damage and increases thrombus formation [172]. Another phenol compound, 4-methylphenol, can inhibit the differentiation of 3T3-L1 preadipocytes into mature adipocytes, induce apoptosis and reduce glucose uptake [173]. In addition, benzoic acid is an aromatic carboxylic acid synthesized by colonic microorganisms by metabolizing dietary aromatic compounds [174]. Plasma benzoic acid levels were found to be elevated in the rat model of polygenic diabetes [174].

Sulfur-containing amino acids such as cysteine and methionine produce sulfides under the action of intestinal bacteria, which are mainly produced by the desulfurization reaction of intestinal bacteria [175]. Studies have found that bacteria such as *Escherichia coli*, *Salmonella, Clostridium* and *Enterobacter aerogenes* in the large intestine can lyse sulfur-containing amino acids [176]. Some bacteria in the human intestine use sulfate as a substrate to produce a large amount of H_2_S, which has various functions such as protecting cells, relaxing blood vessels, regulating blood pressure and reducing heart rate [40]. And H_2_S is also important in the protection of CVDs [177, 178].

In summary, the gut microbiota generates a series of bioactive metabolites to interact with host metabolism [179]. These metabolites mainly include SCFAs, primary and secondary BAs, TMAO, and so on [41]. Among these metabolites, some exert synergistic effects to promote host health by stimulating the parasympathetic nervous system to control glucose metabolism [180]. For example, SCFAs interact with human hormones such as GLP-1 and PYY to effect energy uptake and the development of obesity [41]. SCFAs are beneficial for regulating appetite and energy intake, and preventing the formation of atherosclerosis [181]. Other metabolites, such as TMAO and phenylacetylglutamine (PAGln), may be toxic [182, 183]. For example, TMAO may be proatherogenic to promote the process of thrombosis and atherosclerosis, thereby increasing the risk of cardiovascular events [64]. Patients with CVDs had lower levels of SCFA generation and higher levels of TMAO production due to the change in gut microbiota [184]. Furthermore, TMAO causes vascular endothelial damage and promotes the production of atherosclerotic plaques [182]. TMAO can also increase the release of intracellular calcium ions, enhance the reactivity of platelets, and increase the risk of thrombosis to increase CVDs susceptibility [182]. Besides, PAGln enhances platelet reactivity and thrombosis, leading to CVDs [185]. Taken together, mounting evidence suggests that changes in gut microbiota metabolites are part of the crucial mechanisms by which the gut microbiota regulates the occurrence and development of CVDs.

## The gut microbiota and cardiovascular risk factors

**Fig. 3:**
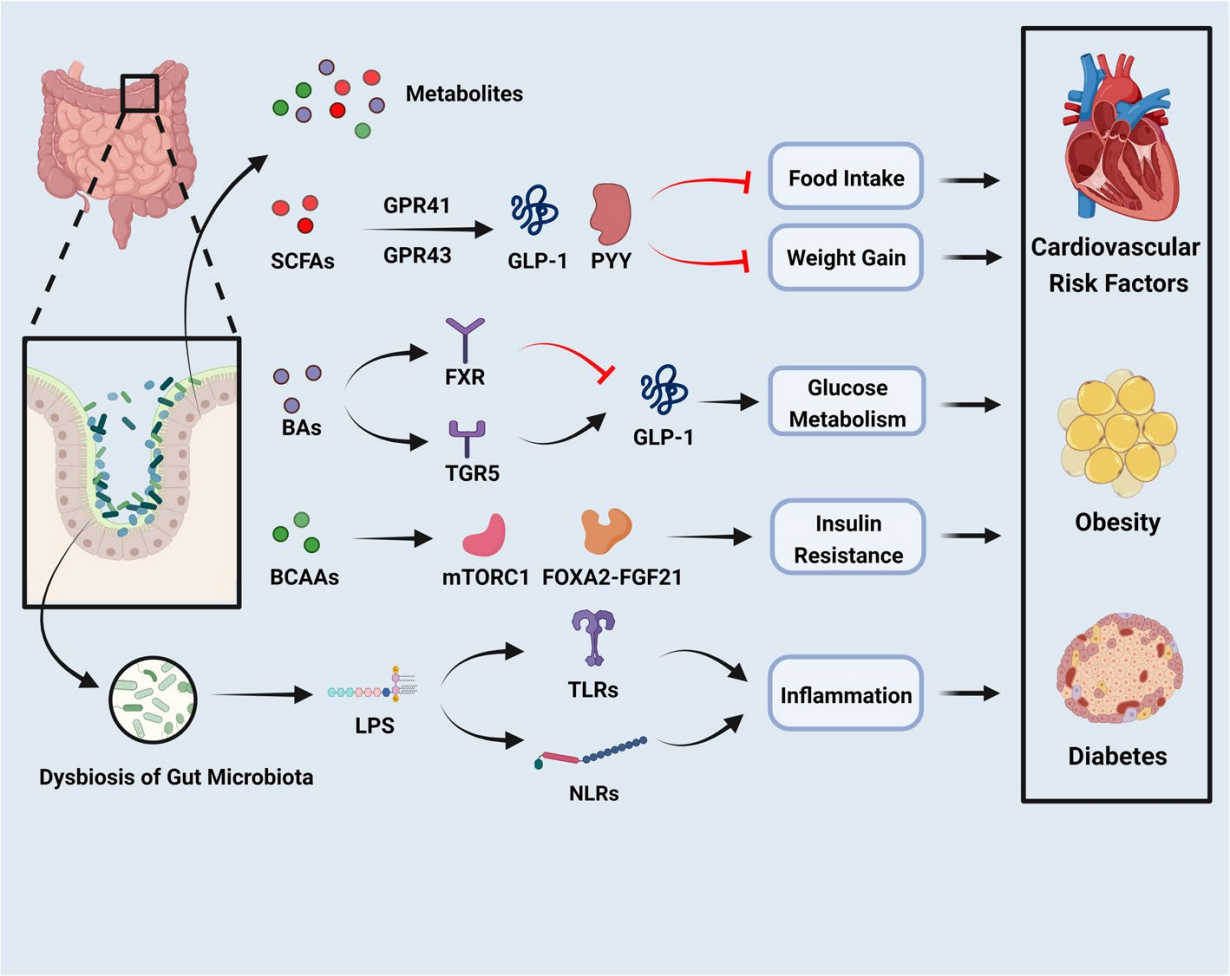
The gut microbiota dysbiosis and its metabolites promote the development of cardiovascular risk factors (created with BioRender.com). The dysbiosis and metabolites of gut microbiota promote the development of obesity and diabetes by increasing myocardial lipotoxicity, diabetic cardiomyopathy, and cardiac insufficiency. SCFAs interact with GPR41/GPR43 to release GLP-1 and PYY, and thus to reduce food intake and weight gain. BAs combine with FXR and TGR5 to regulate glucose metabolism by influencing the release of GLP-1, but their role in obesity and diabetes is controversial. BCAAs regulate mTORC1 and FOXA2-FGF21 to promote insulin resistance. Furthermore, LPS regulates TLRs and NLRs to induce inflammation. Abbreviations: SCFAs: short-chain fatty acids; BCAAs: branched-chain amino acids; BAs: bile acids; LPS: lipopolysaccharide; TGPR, G-protein-coupled receptors; GLP-1: glucagon-like peptide 1; PYY: peptide YY; mTORC1: mammalian target of rapamycin complex 1; FOXA2: forkhead box protein A2; FGF: fibroblast growth factor; FXR: farnesoid X receptor; TGR5: takeda-G-protein receptor 5; TLRs: Toll like receptors; NLRs: Nod-like receptors

The function of the gut microbiota is closely related to the risk of CVDs. Specifically, an impaired mucosal barrier, overactivated inflammation, and immune dysfunction are crucial steps in the development of CVDs triggered by gut microbiota dysbiosis. The structural constituents of gram-negative bacteria, such as LPS, are the main reason for endotoxemia and impaired intestinal mucosal barrier function [186]. It has been reported that LPS is important in the development of cardiometabolic diseases [187, 188]. Moreover, studies showed that the high-fat diet could result in decreased intestinal levels of gram-positive *Bifidobacteria* and increased LPS-containing gut microbiota which further leads to obesity, the main risk factor for CVDs [187, 188]. Importantly, continuous subcutaneous infusion of LPS showed a tendency to cause the change of glucose metabolism and the pattern of weight gain was similar to that seen when taking a high-fat diet [189, 190]. Therefore, intestinal dysbiosis and the corresponding change in metabolites lead to abnormal nutrient metabolism, insulin resistance, and increased adipose tissue storage, which increases the risk of cardiovascular risk factors such as obesity and diabetes [2]. Herein, we take obesity and diabetes as examples to explain the effect of gut microbiota on cardiovascular risk factors (Fig. 3).

### The gut microbiota and obesity

Obesity is a public health concern worldwide, and is attributed to increased energy intake and reduced energy expenditure. With the increasing sedentary lifestyle and diets with high fat as well as high lipid levels, the prevalence of obesity has increased over the last few years with a relatively fast growth rate all over the world [191]. In the latest researches in recent years, gut microbiota dysbiosis has been reported to have a close correlation with the occurrence of obesity [187]. The richness of microbial genes and microbiota load decreased in patients with obesity [192]. Besides, metagenomic analysis suggested increased energy harvest in the gut microbiome of obese mice, which further supported the altered microbiota composition associated with obesity [22, 130]. Except for the change in the composition, the reduced microbial diversity and the metabolites of microbiota are also associated with obesity [193].

#### The dysbiosis of gut microbiota in obesity

Mounting evidence has shown that patients with obesity have a change in the composition of the gut microbiota. In obesity, the increased gut microbiome included the *phylum Molluskum of Firmicutes* [194], *Actinobacteria*, as well as *Firmicutes* and *Bacteroidetes* ratios [195]; the decreased gut microbiome included *Akkermansia*, *Faecalibacterium*, *Oscillibacter*, and *Alistipes* [193]. More importantly, studies indicated that some types of microbes could be regarded as biomarkers for metabolism-related CVDs. For example, it has been reported that *Dorea formicigenerans, Dorea longicatena* and *Collinsella aerofaciens* are associated with obesity, and *Lachnospiraceae* is associated with lipid cardiovascular risk factors [196]. Besides, *Anaeroplasma* and *Haemophilus* are negatively associated with cholesterol and triglycerides and positively associated with HDL [197]. Patients with hyperlipidemia have a lower proportion of *Anaeroplasma* and *Haemophilus* in the gut [197]. Moreover, the ratio of *Firmicutes* to *Bacteroidetes* was also positively associated with obesity [198].

However, considering that most studies about the role of gut microbiota and obesity are cross-sectional studies or association studies, whether obesity disrupts the balance of gut microbiota or the gut microbiota dysbiosis leads to obesity is unknown. Studies have attempted to answer this question by transplanting pathogenic microbiota into germ-free mice. A previous experimental study found that obesity developed after transplantation of penicillin-selected microbiota to germ-free mice [199], which suggested that gut microbiota might be the driving factor for obesity. In our opinion, further studies should focus on exploring the species that play a leading role in contributing to the occurrence of obesity.

#### The metabolites of gut microbiota in obesity

Changes in the composition of gut microbiota increase the risk of obesity, including increasing inflammation, insulin resistance, adiposity deposition, and the capability of harvesting energy from food [200]. Obesity is associated with the changes in serum metabolites [193], mainly including metabolites of SCFAs, BCAAs, and BAs.

##### SCFAs regulate the metabolic hemostasis to promote obesity

SCFAs are metabolites of dietary fibers and resistant starch fermented by gut microbes [201]. The SCFA-producing microbiota are important for cardiovascular health [202]. And SCFAs also directly affect the risk of CVDs, by regulating inflammation, insulin secretion, immune response, intestinal barrier integrity, and energy metabolism [203].

Clinical studies have shown different results regarding the concentration of SCFAs in blood, intestine, and feces in patients with obesity [204]. Some studies reported that patients with obesity had decreased SCFAs-generating gut microbiota and decreased levels of SCFAs in the intestine [204]. Oppositely, other studies suggested that patients with obesity had higher levels of SCFAs in their intestine and feces than healthy participants [205]. They showed that higher fecal SCFAs levels with less efficient SCFAs absorption into blood were associated with obesity and cardiometabolic dysregulation [205]. In a nutshell, these observations suggest that more studies are needed to explore the role of SCFAs in obesity.

SCFAs may prevent obesity by regulating the homeostasis of glucose and lipid metabolism. For instance, acetate decreases appetite and nutrient intake [206]. Propionate activates intestinal gluconeogenesis by a gut–brain neural circuit to promote energy balance [203]. Butyrate provides energy for colon cells, maintains the integrity of intestinal walls, and improves the insulin response, which activates gluconeogenesis to maintain the balance of glucose and energy through the cAMP pathway [203]. And one study indicated that oral butyrate intake prevents obesity and insulin resistance [207]. Furthermore, the role of SCFAs in metabolic hemostasis is related to other factors. Adenosine monophosphate activating protein (AMPK) is a heterotrimeric enzyme that stimulates liver and muscle fatty acid oxidation pathways [208]. SCFAs promote the activity of AMPK and improve its ability to oxidize fatty acids [208]. Last, SCFAs can affect the development of dyslipidemia [209]. SCFAs increase the expression of fasting-induced adipose factor (FIAF), a circulating lipoprotein lipase inhibitor [210], by activating peroxisome proliferator-activated receptor (PPAR-γ) [211, 212], thereby decreasing the activity of lipoproteinases and inhibiting lipolysis in adipose tissues [209]. This process promotes the storage of lipids and the formation of adipose tissue, thus promoting the occurrence of obesity.

##### The association between BAs and the risk of obesity

Several studies have suggested that different BAs profiles are closely related to obesity [213]. It was reported that 12-hydroxylated (12-OH) BAs such as CA and DCA increase obesity susceptibility, while non-12-OH BAs such as CDCA, UDCA and LCA reduce obesity susceptibility [146, 213]. Mice with higher levels of non-12-OH BAs gained weight slowly and had less metabolic disturbance, while the rapidly gaining weight mice had significantly lower levels of non-12-OH BAs such as UDCA [213]. UDCA intake significantly reduced weight gain and metabolic disturbances induced by high-fat diet [213]. Another study also provided evidence that a high-fat diet caused obesity by increasing the levels of 12-OH BAs. In a rat model, high-fat diet caused increases in the total BAs, DCA and taurodeoxycholic acid (TDCA) in plasma and liver tissues, and increases in DCA in intestinal tissues and feces [214]. Therefore, changing the proportion of 12-OH BAs and non-12-OH BAs may have an impact on the risk of obesity [215].

It remains unclear how the gut microbiota and BAs profiles interact with each other and whether the interaction is associated with obesity [147]. Mice fed BAs exhibited obesity and a composition change in the obesity-associated gut microbiota, which is similar to high-fat diet-fed mice [216, 217]. Under a high-fat diet, the concentration of intestinal BAs increases, and the composition of gut microbiota alternates [216]. For example, the increased BAs are the main reason for the marked increase in the abundance of the *Firmicutes* to *Bacteroidetes* ratio [216]. Another study has shown that BAs and a high-fat diet promote the growth of *Bilophila wadsworthia* in mice, and secondary BAs produced by *Bilophila spp.* have been shown to induce obesity [216]. What’s more, it has been reported that the low capacity of microbiota to metabolize Tauro-β-muricholic acid, a primary BA, might increase the risk of obesity and insulin resistance [218, 219]. Therefore, BAs profiles affect the composition of gut microbiota, and gut microbiota also regulates the production and ratio of different BAs to play an important role in obesity susceptibility.

Additionally, studies have also demonstrated that BAs reduce obesity susceptibility by increasing the expression and activity of uncoupling protein 1 (UCP1) and increasing energy expenditure by GLP-1 [220, 221]. For instance, BAs increased the expression of UCP1 in brown adipocytes via TGR5 to enhance the release of GLP-1 [222]. Thus, there is an interrelated complex relationship between BAs and obesity. It is essential to assess the role of changes in the composition of BAs in the downstream signaling pathways of the host receptors to gain a greater mechanistic understanding of obesity susceptibility.

##### BCAAs promote insulin resistance to induce obesity

Patients with obesity had increased BCAAs that contribute to the development of obesity-associated insulin resistance [165, 223]. In fact, it is controversial about the mechanism of BCAAs-related insulin resistance [224]. Insulin resistance induced by high-fat and supplemental BCAAs feeding was accompanied by chronic phosphorylation of mammalian target of rapamycin (mTOR), c-Jun N-terminal kinases (JNK), and insulin receptor substrate (IRS)1 _Ser307_ [224]. BCAAs inhibit white fat browning and promote obesity-related metabolic disorders [225]. Some studies have suggested that the role of BCAAs is related to mammalian target of rapamycin complex 1 (mTORC1) [226]. And BCAAs are potent mTORC1 agonists, and a lifelong BCAAs-restricted diet downregulates mTORC1 signaling, reduces frailty, and prolongs lifespan in wild-type male mice [227]. Nevertheless, another study found that the metabolic effects of a low BCAAs diet did not require inhibiting the expression and activity of hepatic mTORC1 [227].

### The gut microbiota and diabetes

In addition to the clear relationship between gut microbiota and the occurrence of obesity, gut microbiota has also been associated with the development and prognosis in patients with diabetes [125]. Diabetes are associated with a relative or absolute insufficiency of insulin, or insulin resistance, which results in metabolic disturbance of carbohydrates, lipids, and proteins [228]. In China, the number of diabetic patients has reached 114 million, accounting for 1/3 of the total number of diabetic patients all around the world [229]. Type 2 diabetes (T2D) accounts for more than 95% of the total diabetes population [230]. Recently, researchers have pointed out that gut microbiota may play an important role in the occurrence and development of T2D [125, 231]. Thus, exploring the relationship between gut microbiota and T2D may provide new ideas for clinical research and treatment.

#### The dysbiosis of gut microbiota in diabetes

The changes in microbiota and the increased abundance of facultative pathogens are already present in patients with new-onset diabetes, suggesting that microbiome instability is associated with cardiovascular risk factors [232]. *Bacteroidetes* are detected in children genetically predisposed to type 1 diabetes (T1D) [233]. Regarding T2D, patients were characterized by a decrease in several butyrate-producing bacteria and an increase in many opportunistic pathogens [234]. For example, patients with T2D have reduced *Clostridium butyricum* in the gut, a main butyrate-producing bacteria [235, 236]. Lower concentrations of butyrate producing microbiota have been observed in fecal samples of patients with T2D [22]. Moreover, *Lactobacillus* is positively correlated with fasting blood glucose and glycated hemoglobin levels, while *Clostridium* is negatively correlated with fasting blood glucose and glycated hemoglobin levels [237]. Patients with T2D have significantly higher levels of *Lactobacillu*s in fecal samples than healthy individuals [238]. These data suggest a strong association between gut microbial dysbiosis and the pathology of diabetes.

#### The metabolites of gut microbiota in diabetes

Multiple metabolites including SCFAs, BAs, and BCAAs, have been postulated to link the potential association between altered gut microbiota and T2D. Specific species such as *Faecalibacterium prausnitzii* have been observed to produce anti-inflammatory cytokines and chemokines, thus alleviating inflammation and increasing insulin sensitivity [239]. And some intestinal bacteria from other genera have been reported to produce pro-inflammatory cytokines, which may increase the development of insulin resistance [240]. In short, the dysbiosis of gut microbiota would lead to the damaged function of β-cell and islet chronic inflammatory response by changing the component of SCFAs, BCAAs, and BAs, thereby affecting glucose and fat metabolism, eventually leading to the occurrence of diabetes [241].

##### SCFAs regulate glucose metabolism to affect diabetes

Insufficient dietary fiber intake is considered to be an important risk factor for diabetes [242]. The decrease in the production of SCFAs counts for an important reason here [243]. Modulation of dietary SCFAs is thought to reshape the gut microbiota in diabetes and ameliorate diabetes [125]. SCFAs increase fatty acid oxidation and energy expenditure to decrease the risk of suffering from T2D [234]. Of note, T2D patients have lower levels of butyrate-generating bacteria [243]. Butyrate could promote the secretion of postprandial insulin; and the increase in fecal propionic acid was associated with an increase in T2D risk [243]. In oral glucose tolerance test, a high butyrate-producing microbiota was associated with an improved insulin response (indicating improved β-cell function), and butyrate concentration was directly related to postprandial insulin sensitivity [244]. Besides, in signal transduction regulation, butyrate could also inhibit HDAC expression in juvenile diabetic rats to modulate the p38/ERK/MAPK signaling pathway, which ultimately prevents β-cell apoptosis and improves glucose homeostasis [245].

SCFAs influence blood glucose levels [246] and are associated with the regulation of intestinal gluconeogenesis [203]. Two SCFAs, propionate and butyrate, promote the secretion of postprandial insulin and activate intestinal gluconeogenesis control [203]. Butyrate activates the gene expression of intestinal gluconeogenesis by the cAMP pathway, while propionate is a substrate for intestinal gluconeogenesis, and approximately 50% of the preferred precursor for gluconeogenesis is propionate [203]. Bacterial fermentation of dietary fiber produces large amounts of succinate, which improves blood glucose by activating intestinal gluconeogenesis control [203]. Elevated postprandial plasma butyrate concentration is associated with increased abundance of *Intestinimonas butyriciproducens* and *A. muciniphila* [244, 247]. Therefore, regulating the levels of SCFAs production may be a promising approach to the regulate the development of diabetes.

##### The role of different composition of BAs in diabetes

BAs are steroid carboxylic acids mainly derived from cholesterol through the action of CYP7A1, and their main function is to digest and absorb lipids and fat-soluble vitamins in the small intestine [248]. The gut microbiota can also affect health by modulating the metabolic levels of total BAs, DCA and LCA, either by metabolizing bile salts or modulating downstream signaling pathways of BAs [153]. Most gut microbiota have BSH activity [249]. For example, *Firmicutes* have CYP7A1, which most intestinal microorganisms do not have [250]. *Bacteroides* can oxidize, epimerize and esterify BAs at the same time [234].

The gut microbiota can affect the occurrence of diabetes by affecting the composition and metabolism of BAs, as well as their binding to FXR and TGR5 receptors. An excess of secondary BAs produced by dysregulated gut microbiota can stimulate intestinal parietal cells to secrete a large amount of serotonin, thereby increasing blood glucose levels [251]. In patients with diabetes, changes in gut microbiota composition may alter the ratio of primary BAs to secondary BAs [252]. However, from the impaired fasting glucose level to impaired glucose tolerance, and finally to T2D, the specific changes in gut microbiota and BAs pools at different stages of this process have not been fully studied.

Previous results have indicated that the metabolic regulation of BAs may be associated with T2D but the specific changes in BAs and the related mechanisms remain to be studied [253]. It is also unclear how the different types of BAs change in patients with T2D. A study also suggested that patients with T2D had no change in plasma total BAs, but had an increase in DCA and a decrease in CDCA in plasma [254]. Another study found that the plasma total BA concentration in patients with T2D was not significantly different from that of healthy people [255]; however, the concentration of DCA in the plasma of patients with T2D was significantly higher than that of healthy controls [255]. The mechanism may be that FXR and TGR5 are inhibited during insulin resistance, and the most effective natural ligands of the receptors, DCA and CA, are compensatorily increased [255]. Besides, circulating BAs levels including DCA, TCA, TDCA, and glycodeoxycholic acid, are positively associated with the risk of T2D, while taurohyodeoxycholic acid is negatively associated with diabetes [256]. This may be due to the fact that hydrophobic BAs isoforms (such as DCA) have been shown to be involved in inflammation and endoplasmic reticulum stress with glucose dysregulation, and hydrophilic BAs isoforms (such as tauroursodeoxycholic acid) have been shown to prevent inflammation and enhance insulin sensitivity [257].

Postprandial BAs profiles are correlated with postprandial lipids, waist circumference, and body mass index (BMI), suggesting that changes in BAs metabolism in response to a high-energy diet may reflect healthy or unhealthy metabolic phenotypes [258]. In fact, the gut microbiota can regulate glucose metabolism by regulating the interaction between BAs and FXR and TGR5 signaling [112]. In the regulation of signaling pathways, primary BAs can stimulate FXR on pancreatic β-cells in order to promote insulin release. Then, the activation of FXR signaling can stimulate ileal secretion of FGF19. FGF19 has insulin-like effects that regulates BAs synthesis by reducing CYP7A1 expression, inhibiting glucose production, and inducing glycogen synthesis. And FGF19 can inhibit the phosphorylation of cAMP response element binding protein (CREB), thereby reducing hepatic gluconeogenesis, promoting hepatic glycogen production, and inhibiting GLP-1 release [259–261]. In addition, FGF19 can also activate the RAS/ERK pathway which promotes the phosphorylation of glycogen synthase kinase (GSK) α and β, and enhances the activity of glycogen synthase, which can increase hepatic glycogen synthesis [262].

Nevertheless, the role of FXR in diabetes is controversial. Although study has reported that upregulating FXR in diabetic mice can significantly improve hypercholesterolemia [263], other study found that inhibition of intestinal FXR can reduce hepatic gluconeogenesis, promote the secretion of GLP-1, and reduce body weight [264]. Therefore, whether upregulating or inhibiting FXR signaling is an innovative approach in the control of blood glucose in patients with T2D needs further research.

In addition, secondary BA can stimulate TGR5, promoting the release of GLP-1 from enteroendocrine cells [220]. And activation of TGR5 further improves glycemic control and energy homeostasis [265, 266]. Moreover, BAs inhibit the activation of the NLRP3 inflammasome through the TGR5-cAMP- protein kinase A (PKA) axis, and block LPS-induced systemic inflammation as well as T2D-related inflammation [267, 268]. Taken together, it is necessary to carry out dynamic tracking research on intestinal microbiota and BAs pools, so as to facilitate the development of targeted therapy for downstream signals of BAs.

##### BCAAs promote insulin resistance to trigger diabetes

Another important mechanism of gut microbiota related to diabetes is the modulation of BCAAs. Evidence indicates that gut microbiota can change the decomposition of protein as well as the level of BCAAs in plasma [269]. BCAAs and their metabolites are the most significant factors distinguishing normal from abnormal metabolism [270]. Plasma BCAAs levels of T2D patients are higher than those with normal blood glucose, and BCAAs levels are positively correlated with the homeostasis model-assessed insulin resistance index [271]. Studies have suggested that elevated blood BCAAs concentrations are associated with an increased risk of T2D and insulin-resistance, and elevated BCAAs caused by dysmetabolism are important risk factors for T2D [272, 273]. Additionally, BCAAs also serve as biomarkers that monitor the treatment effect of T2D [274].

It is well investigated that BCAAs have a significant positive correlation with blood glucose, blood lipids, and the insulin resistance index, and can be used as potential biomarkers for the early prediction of diabetes [275]. One study indicated that three BCAAs (leucine, isoleucine, and valine), and two aromatic amino acids (phenylalanine and tyrosine) were significantly increased 10 years before the onset of diabetes [275]. The increased BCAAs-induced insulin resistance was associated with the presence of *Prevotella copri* and *Bacteroides vulgatus* [269]. The major groups of gut bacteria that biosynthesize BCAAs are *Prevotella copri* and *Bacteroides vulgatus,* and the insulin resistance caused by elevated BCAAs levels is associated with the presence of these two bacteria [269]. Of note, feeding *Prevotella* to mice indicated that *Prevotella* could induce insulin resistance, resulting in increased BCAAs levels as well as glucose intolerance [269].

The mechanism by which BCAAs induce insulin resistance is still not fully understood. The rise of BCAA levels is found to regulate glycolipid metabolism, cause insulin resistance, and increase the risk of T2D [272]. Other animal experiments have shown that elevated BCAAs are transferred to skeletal muscle, and are interfered with lipid metabolism, which causes the accumulation of lipid metabolites and ultimately leads to skeletal muscle insulin resistance [276]. Additionally, BCAAs are the key regulatory factor of mTORC1. It activates mTORC1 and ribosomal protein S6 kinase beta-1 (S6K1) by inducing the phosphorylation of IRS1, blocking the insulin signaling pathway, and eventually causing insulin resistance [277]. However, 2 other studies have shown that BCAAs may exert their functions without mTORC1 [119, 278]. One study reported that the metabolites of BCAAs, 3-hydroxy isobutyrate, could cause insulin resistance by promoting skeletal muscle sprouting [278]. Another study reported that a diet rich in BCAAs can cause overeating, obesity, and shortened lifespan, which was not related to the mTOR signaling pathway in the liver but is mainly due to hyperphagia [119].

It is currently controversial whether supplementation with BCAAs would increase circulating BCAAs, thereby increasing the risk of diabetes [279]. And reducing BCAAs intake promotes fat mass loss and insulin sensitivity in mice with obesity [280]. A high BCAAs diet causes excessive feeding, mainly due to excessive appetite. Adding threonine to balance the composition of amino acids in the diet can inhibit food intake [119]. Nevertheless, some studies have found that supplementary BCAAs, such as isoleucine and leucine, can improve insulin resistance and reduce weight to lower the risk of obesity and diabetes [281–283]. The inconsistency may be due to the individual state of energy excess or energy deficiency. In terms of an excessive energy state, such as in patients with obesity or diabetes who have an impaired ability to degrade BCAAs, BCAAs and related metabolites accumulate in circulation to reduce insulin sensitivity and cause insulin resistance [284]. In most elderly people with inadequate energy or athletes who maintain good fitness habits, BCAAs can improve the form of metabolism [285]. In these people, BCAAs decomposition metabolism is increased by regulatory factors such as peroxisome proliferator-activated receptor-α coactivator (PGC-1α) and PPARα, so that BCAAs levels are maintained in a steady state, which ultimately promotes glucose intake and insulin sensitivity [285]. This was supported by the study of Newgard, who pointed out that dietary intake of BCAAs might interact with a high-fat diet to induce abnormal metabolic phenotypes such as insulin resistance [224]. Mice fed BCAAs alone did not develop insulin resistance. However, when mice were fed a high-fat diet supplemented with BCAAs, they developed insulin resistance [224]. This study suggested that BCAAs only lead to insulin resistance in the presence of excess energy intake [224]. Therefore, more studies are needed to explore the supplementation with BCAAs and insulin resistance as well as the development of cardiovascular risk factors.

## The role of the gut microbiota in CVDs

A healthy gut microbiota can resist the invasion of foreign pathogenic microorganisms by constructing a mucosal barrier and maintain the stability of the intestinal environment as well as microecological balance [286]. When the species, proportion and number of gut microbiota are normal, the original gut microbiota can produce colonization resistance, preventing the reproduction of pathogenic bacteria and settlement [287, 288].

However, under certain circumstances, pathobionts can translocate from the gut mucosa to the systemic circulation, leading to systemic inflammation and deleterious effects in CVDs progression [289]. It has been found that oral bacteria, including *Streptococcus* and *Vernonella*, have increased ectopic colonization in the intestines of patients with T2D, coronary artery disease (CAD), and inflammatory bowel diseases [290].

In recent years, many researches demonstrated that gut microbiota could promote the development of cardiovascular risk factors and then further promotes the development of CVDs. After all, one of the mechanisms by which gut microbiota dysbiosis is associated with CVDs is the impaired intestinal mucosal barrier function and increased intestinal permeability under the interaction between LPS and host receptors [291–293].

In fact, LPS plays an important role in mediating inflammatory responses in vivo through TLRs [294]. TLRs are important mediators of the innate immune system. Previous studies have shown that atherosclerotic arteries express TLRs, and the activation of TLRs, specially TLR2 and TLR4, has a certain influence on atherosclerosis [295]. Furthermore, LPS induces endothelial cell injury, stimulates the oxidative metabolism of monocytes, and causes LDL oxidation in order to induce the transformation of macrophages into foam cells, which further promote the development of atherosclerosis [296]. LPS binds to its binding protein to form the complex and is recognized by TLR4 on the surface of immune cells, followed by neutrophil infiltration and the accumulation of inflammatory factors (TNF-α, IL-1, IL-27, etc.), which increases the risk of atherosclerosis [297, 298]. LPS can activate the TLR4-mediated pathway including the nicotinamide adenine dinucleotide phosphate (NADPH)/reactive oxygen species (ROS)/endothelial nitric oxide synthase (eNOS) and MAPK/NF-κB pathways, leading to endothelial dysfunction and vascular inflammation [69]. Notability, the concentration of plasma LPS has been reported to be positively correlated with hypertension [56]. And the dysbiosis of gut microbiota and oxidative stress can promote the oxidation of LDL to oxLDL, which can inhibit the expression of eNOS, further leading to vasoconstriction and promoting the occurrence of hypertension [299–301].

Despite these important advances in understanding the underlying mechanisms and signals in modulating atherosclerosis and hypertension [302], the effect and mechanism of LPS in CAD have not been fully explored. However, at least in some recent studies, an increasing potential for LPS biosynthesis in the microbiota in patients with CAD has been reported [303, 304]. The increased production of LPS biosynthesis in the microbiota has been reported to predict adverse cardiovascular events in patients with CAD, which may increase intestinal permeability [304]. Following antibiotic treatment, gut bacterial translocation, LPS-induced systemic inflammation, and cardiomyocyte injury in MI mice were alleviated [304]. However, in a separate study, the circulating markers of gut related inflammation LPS binding protein and soluble CD14 were modulated by neither n-3 PUFA supplementation nor diet intervention [305]. In addition to the LPS of gut microbiota affecting the occurrence and development of CVDs, the dysbiosis of gut microbiota and the changes of its metabolites are also closely associated with CVDs. Hence, further studies are needed to clarify the associations between gut microbe-derived LPS and CVDs, aiming to develop promising targeted therapies for CAD. Next, we further elaborated the role of gut microbiota in common CVDs by disease classification.

### The gut microbiota and hypertension

**Fig. 4:**
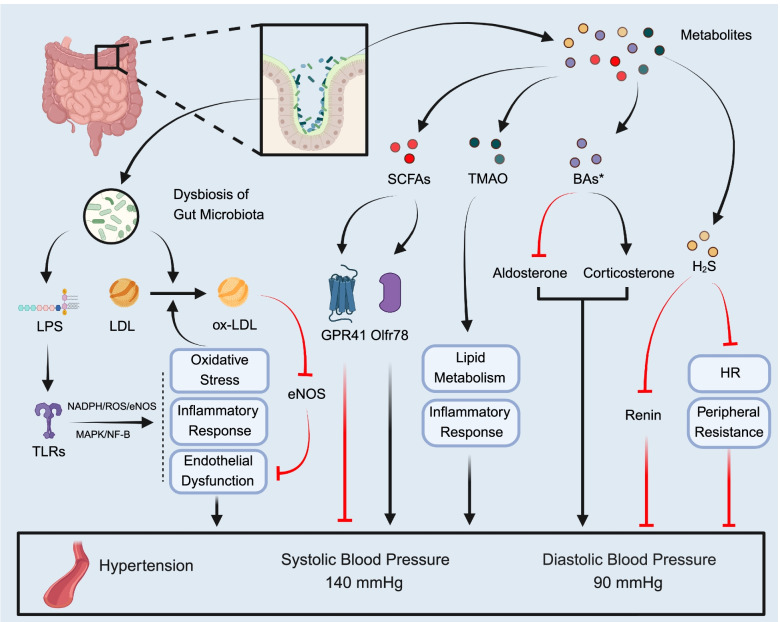
The mechanism of gut microbiota increasing the risk of hypertension (created with BioRender.com). Hypertension is diagnosed in adults with systolic blood pressure ≥ 140 mmHg or diastolic blood pressure ≥ 90 mmHg. The dysbiosis and metabolites of gut microbiota can influence the course of hypertension. Abbreviations: SCFAs: short-chain fatty acids; TMAO: trimethylamine N-oxide; BAs: bile acids; LPS: lipopolysaccharide; TLRs: Toll like receptors; GPR41: G protein-coupled receptor 41; Olfr78: olfactory receptors 78; HR: heart rate; LDL: low density lipoprotein; ox-LDL: oxidized low-density lipoprotein; eNOS: endothelial nitric oxide synthase. *The regulatory effect of BAs on blood pressure is still controversial, and the figure shows only one possible mechanism

The pathogenesis of hypertension is complicated and influenced by environmental and genetic factors [306]. Effective antihypertensive therapy can reduce target organ damage and further improve quality of life [307]. Studies showed that the gut microbiota could produce a large number of metabolites through the absorption and decomposition of nutrients, which in turn affect the occurrence and development of hypertension (Fig. 4) [308, 309]. Therefore, reducing the dysbiosis of gut microbiota and regulating their metabolites may have the potential to lower the blood pressure [310].

#### The dysbiosis of gut microbiota in hypertension

The most significant characteristic of gut microbiota in hypertensive patients is the decrease in microbial diversity, richness and the uneven distribution [309]. Researchers conducted 16S amplicon sequencing on fecal samples from patients with hypertension, and used Bayesian network analysis to find the relationship between blood pressure and the abundance of gut microbiota. The results showed that changes in blood pressure could affect bacterial abundance [311]. Moreover, Nathalia Santos Magalhães et al. reviewed the changes in gut microbiota in hypertensive patients, in which *Klebsiella*, *Desulfovibrio* and *Prevotella* increased, while *Blautia*, *Butyrivibrio*, *Clostridium*, *Enterococcus*, *Faecalibacterium*, *Oscillibacter*, *Roseburia*, *Bifidobacterium*, and *Lactobacillus* decreased [312].

In addition, the abundance of gut microbiota is associated with salt sensitivity in hypertension. It was reported that moderate high salt stimulation reduced the survival of *Lactobacillus* in the intestine and increased T_H_17 cells, which could lead to an increase in blood pressure [313]. Besides, gut microbiota has been found to promote angiotensin II-induced hypertension by supporting monocyte chemoattractant protein 1/IL-17 driven vascular immune cell infiltration and inflammation [314]. Therefore, the dysbiosis of gut microbiota may have an impact on the development of hypertension.

#### The metabolites of gut microbiota in hypertension

Dysbiosis of gut microbiota can further lead to changes in metabolites, which are complex in regulating blood pressure [315]. So far, SCFAs and TMAO are the main research objects, and BAs and H_2_S have also been partially reported [159, 316].

##### SCFAs regulate the blood pressure by different pathways

SCFAs are well reported to regulate blood pressure by GPR41 and Olfr78 [317, 318]. It was reported that the systolic blood pressure of GPR41 knockout mice was higher than that of wild-type mice [319]. Olfr78 can participate in the activation of the sympathetic nerve, the formation of hypertension and the increase in carotid body activity [318]. Besides, the plasma renin activity in Olfr78-KO mice was lower than that in wild type mice, which may be induced by high levels of SCFAs activation [317, 320]. In short, gut microbiota may play a crucial role in the regulation of hypertension through SCFAs-mediated mechanisms, and SCFAs have the potential to be a therapeutic target for hypertension.

##### TMAO increases the risk of hypertension

The concentration of circulating TMAO is positively correlated with the risk of hypertension and people with high concentrations of circulating TMAO are more likely to suffer from hypertension [321, 322]. In animals with normal blood pressure, TMAO has no direct effect on blood pressure, but can prolong the action time of hypertension induced by angiotensin II [323]. However, the specific molecular mechanism of the effect of TMAO on blood pressure is not completely clear. Previous research revealed that TMAO increased blood pressure by upregulating inflammatory gene expression and pathways in human aortic endothelial cells and vascular smooth muscle cells [324]. In fact, the effect of TMAO on blood pressure is mainly related to lipid and glucose metabolism and inflammation, which is similar to the pathogenesis of atherosclerosis caused by TMAO [240]. After all, persistent hypertension can lead to endothelial cell damage and lipid deposition, which can lead to atherosclerosis [325]. However, the specific molecular mechanism of the effect of TMAO on blood pressure needs further exploration.

##### BAs and H_2_S play roles in the development of hypertension

At present, there are few studies on the effect of BAs on blood pressure, and results are not completely consistent. One study found that BAs could decrease aldosterone and increase corticosterone in blood vessels, which could induce hypertension [326], but another study suggested that BAs acted directly on the vascular bed and weakened the response of blood vessels to norepinephrine [327]. Of note, a recent study showed that BAs supplementation significantly reduced blood pressure in rats susceptible to spontaneous hypertensive stroke [328]. Therefore, there is a possible relationship between BAs and the formation of hypertension, but whether they play a beneficial or harmful role needs further exploration.

Besides, the gut microbiota can also produce H_2_S to participate in oxidation regulation, inflammation and other processes [329]. It has been found that colon-derived H_2_S can lower blood pressure [177]. RAS, in particular, is an essential mechanism in the pathogenesis of hypertension [330]. H_2_S has been reported to inhibit the activity of renin by reducing the synthesis and release of renin, which has potential therapeutic value for renovascular hypertension [331]. Moreover, H_2_S may reduce blood pressure by dilating peripheral blood vessels and reducing heart rate [177, 178].

Taken together, the etiology of hypertension is complex, the composition of gut microbiota is diverse, and the dysbiosis of gut microbiota will further affect their metabolites [332]. Although the specific molecular mechanism between the metabolites and blood pressure is not completely clear, it is expected to enrich the existing treatment of hypertension by regulating gut microbiota [333].

### The gut microbiota and atherosclerosis

**Fig. 5:**
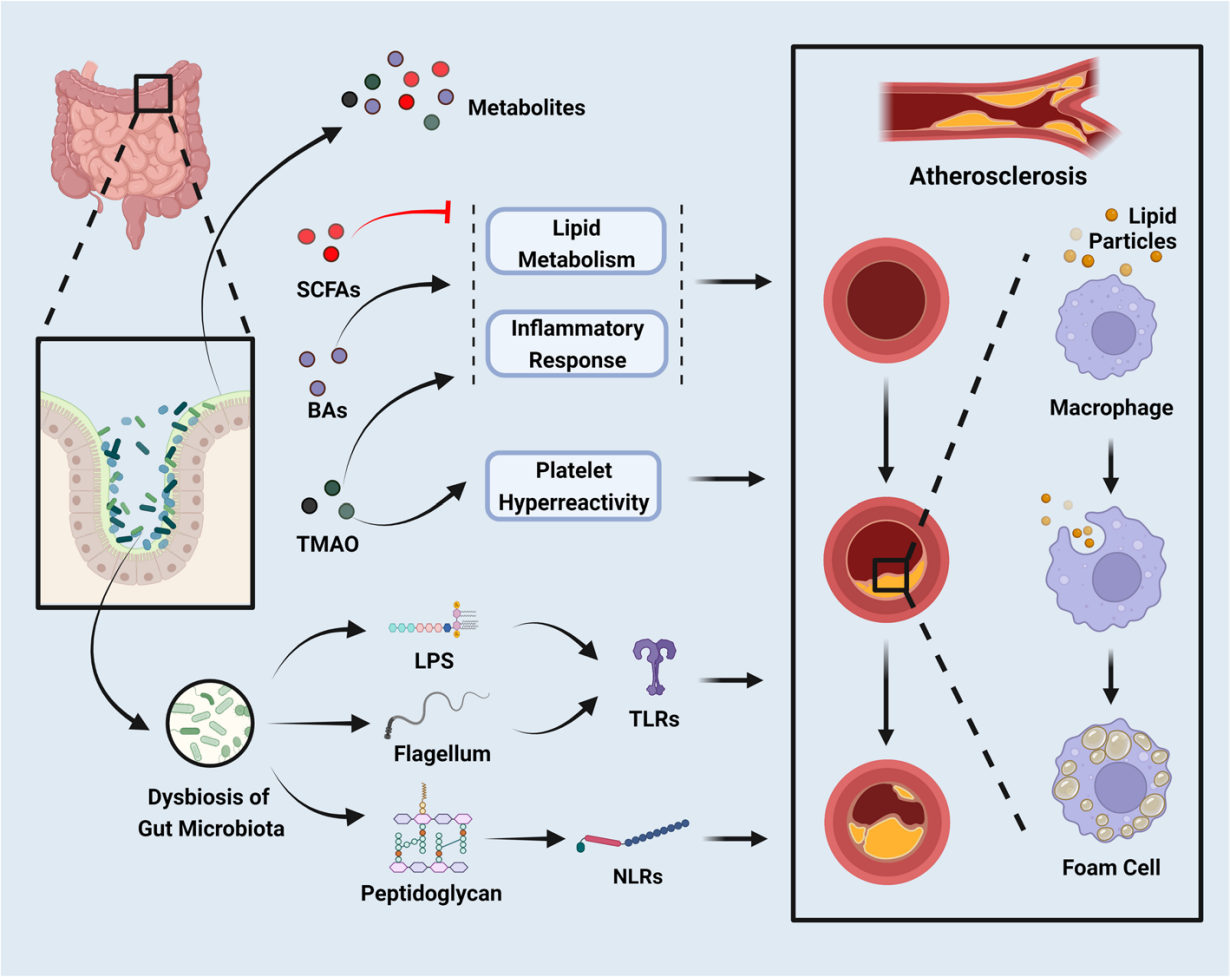
The relationship between gut microbiota and atherosclerosis (created with BioRender.com). Due to the accumulation of cholesterol in the vascular wall, macrophages engulf lipid particles and convert them into foam cells, further aggravating atherosclerosis. The dysbiosis and metabolites of gut microbiota can influence the course of atherosclerosis. In general, SCFAs are beneficial for improving atherosclerosis, while BAs and TMAO may aggravate the progression of atherosclerosis. Abbreviations: SCFAs: short-chain fatty acids; TMAO: trimethylamine N-oxide; BAs: bile acids; LPS: lipopolysaccharide; TLRs: Toll-like receptors; NLRs: Nod-like receptors

Atherosclerosis is the pathological basis of various CVDs, and seriously harms human health [334]. The pathogenesis of atherosclerosis is based on the accumulation of lipids in the intima of the most frequently involved arteries, leading to the proliferation of fibers and calcium deposition, gradually thickening and hardening the walls of blood vessels [335]. This process is closely associated with the inflammatory response [336, 337]. Of note, there is a serious dysbiosis between the composition of gut microbiota and interspecific relationships in individuals with atherosclerosis [338]. And the metabolites of gut microbiota can also further affect atherosclerosis [94, 339] (Fig. 5).

#### The dysbiosis of gut microbiota in atherosclerosis

As mentioned above, an intact intestinal epithelium can effectively prevent the translocation of bacteria to the intestine [340]. Normal gut microbiota can maintain the structural integrity of the intestinal mucosal barrier [341]. It has been reported that intestinal metagenomic changes in individuals with atherosclerosis are associated with the inflammatory response [338, 342]. It has been found that bacteria of the *Collinsella* genus were enriched in atherosclerotic patients, while SCFA-producing bacteria, such as *Roseburia* and *Eubacterium*, were enriched in healthy individuals [342, 343]. In fact, impaired intestinal barrier integrity due to dysbiosis in the gut microbiota is considered a risk factor for atherosclerosis [344]. When the gut microbiota is disordered and the intestinal epithelial barrier is damaged, immune cells recognize a variety of bacterial components through pattern recognition receptors to recognize highly conservative pathogen-associated molecular patterns, leading to systemic and tissue-specific inflammation [345].

#### The metabolites of gut microbiota in atherosclerosis

##### SCFAs prevent the formation of atherosclerosis

It is known that SCFAs can reduce intestinal inflammation, prevent pathogens from invading, and maintain barrier integrity [41]. For example, acetic acid has been found to ameliorate chronic inflammation in atherosclerosis [346, 347]. Besides, it has been reported that propionic acid can improve atherosclerosis by immune-dependent regulation of intestinal cholesterol metabolism [348]. In addition, butyric acid can improve atherosclerosis by decreasing NF-κB activation, reducing macrophage adhesion and migration, and alleviating inflammation [349]. Another study found that butyric acid can also improve the transcription of apolipoprotein A-I in the liver, which helps improve the function of HDL and prevent atherosclerosis [350]. In brief, SCFAs can be a potential therapeutic target for atherosclerosis by regulating lipid metabolism and reducing inflammation [351].

##### TMAO promotes endothelial dysfunction and regulates lipid metabolism to increase atherosclerosis

TMAO actively participates in the development and progression of atherosclerosis, which can lead to endothelial dysfunction, affect platelet activation, as well as participate in thrombus formation [94]. The inflammatory response is one of the key factors in atherosclerosis caused by TMAO [94, 352]. It has been reported that TMAO can promote vascular inflammation and endothelial dysfunction by activating NLRP3 inflammatory bodies, and the specific molecular mechanism may be the inhibition of the Sirtuin 3-superoxide dismutase 2-mitochondrial ROS pathway and the activation of the ROS-thioredoxin-interactive protein axis [353, 354]. Furthermore, TMAO can enhance the adhesion ability of monocytes and promote atherosclerosis by activating the protein kinase C/NF-κB/vascular cell adhesion molecule-1 pathway [352].

Additionally, TMAO can cause atherosclerosis by affecting lipid metabolism. For example, TMAO inhibits the synthesis of BAs and accelerates the formation of aortic lesions in ApoE^−/−^ mice, and the specific molecular mechanism is involved in the activation of FXR and small heterodimer partners [355]. TMAO can also upregulate CD36 expression, class A1 scavenger receptor, and cholesterol migration related gene ATP-binding cassette transporter A1 in macrophages, resulting in cholesterol accumulation in macrophages [356–358]. Moreover, platelets, which play an important role in the occurrence and development of atherosclerosis, are also regulated by TMAO. Besides, TMAO promotes platelet hyperreactivity and thrombosis by increasing intracellular Ca^2+^ release [359]. In a nutshell, TMAO can be a potential target for the prevention and treatment of atherosclerosis [360].

##### The controversial role of BAs in atherosclerosis

As we known, BAs also play an important role in gut microbiota and cholesterol excretion [144]. Cholesterol is metabolized in the liver to produce primary BAs, which are absorbed at the end of the ileum and then transported to the liver [361]. BAs mainly bind to FXR and TGR5 to exert biological roles in the progression of atherosclerosis [362, 363]. Notability, the regulation of FXR and TGR5 can also influence the progression of atherosclerosis [364]. It was reported that the loss of FXR was associated with increased atherosclerosis in ApoE^−/−^ mice [365]. The clinical application of FXR agonists also showed a regulatory effect on blood lipids [366]. However, some studies have shown that the inhibition of FXR can promote BAs metabolism and improve atherosclerosis [367]. We proposed that different types of BAs may have opposite effects on FXR, and the final regulation of atherosclerosis depends on their comprehensive effect. As for TGR5, it has been reported to the activation of TGR5 could reduce the area of atherosclerotic plaques, reduce inflammation in plaques, and inhibit phagocytosis of oxLDL by macrophages [368, 369]. In addition, it has been found that an FXR/TGR5 double agonist (INT-767) can significantly reduce atherosclerotic plaques in ApoE^−/−^ and LDLR^−/−^ mice by inhibiting the expression of NF-κB [370]. Given that the conclusions of FXR/TGR5 regulating atherosclerosis are not completely consistent, more high-quality studies are needed to further clarify the regulatory role of BAs targeting FXR/TGR5 in atherosclerosis.

At present, the main clinical treatments for atherosclerosis are lipid-lowering drugs, antiplatelet drugs and anti-inflammatory drugs, but these treatments may not have ideal effects on some patients [336, 371]. Therefore, finding new drug targets and developing methods for the treatment of atherosclerosis are of great significance to reduce the morbidity and mortality of CVDs, while targeted therapy for gut microbiota and regulation of gut microbiota and their metabolites, may be a good choice.

### The gut microbiota and CAD

**Fig. 6:**
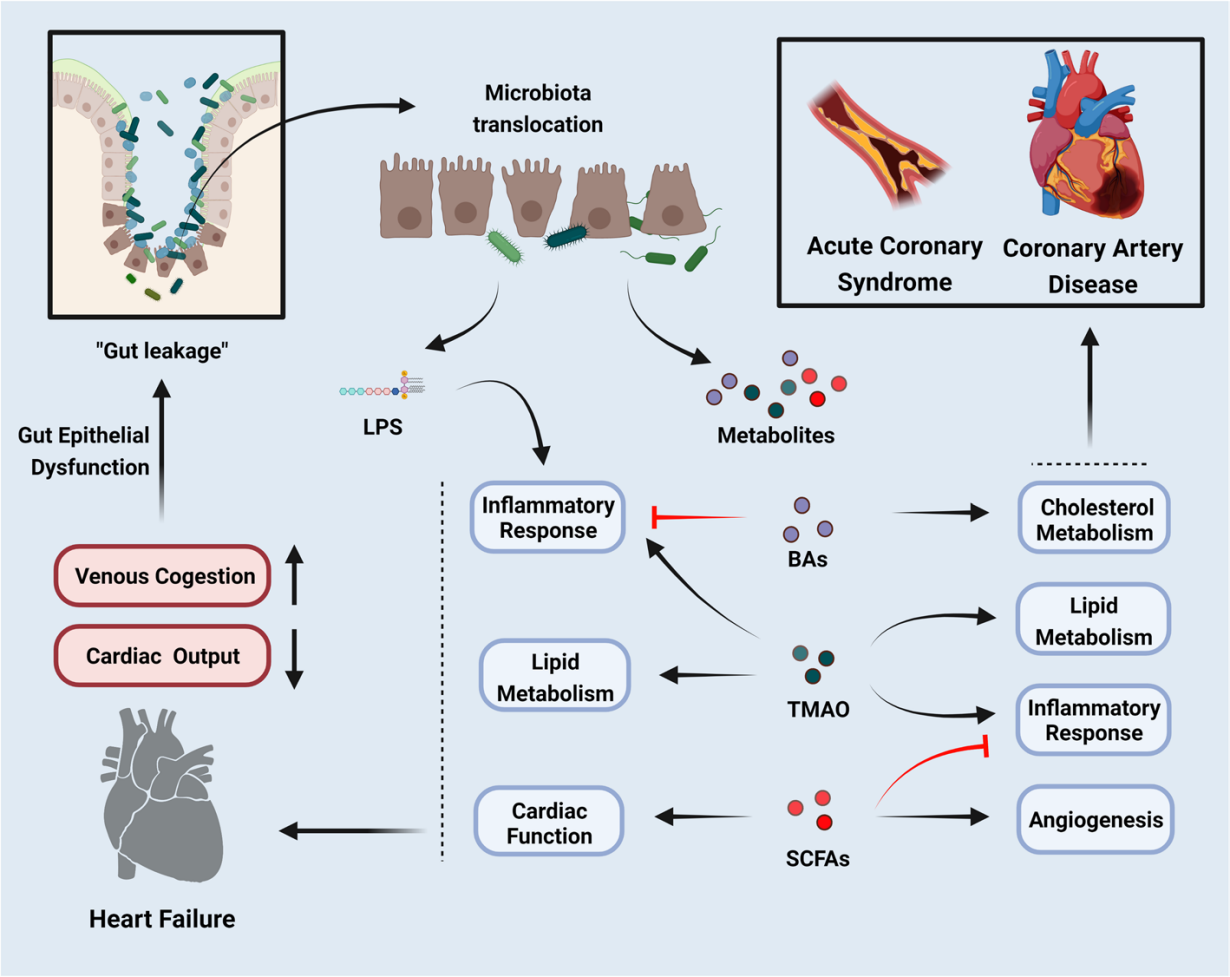
The gut leakage promotes the formation of coronary heart diseases and heart failure (created with BioRender.com). Dysbiosis of gut microbiota triggers gut leakage, which increases microbiota translocation, resulting in inflammation and abnormal metabolism. The gut microbiota, as a metabolic filter, also converts nutrients to (microbial-associated) metabolites, including BAs, TMAO, SCFAs, and so on, which affect the progression of coronary heart diseases and heart failure. For instance, SCFAs play a protective role by inhibiting the inflammatory response and improving angiogenesis. All these metabolites may promote venous congestion and decrease cardiac output, eventually leading to heart failure. Abbreviations: BAs: bile acids; SCFAs: short-chain fatty acids; TMAO: trimethylamine N-oxide; LPS: lipopolysaccharide

CAD, one of the major CVDs, has remained the leading cause of mortality worldwide in decades [372, 373]. The pathogenesis of CAD is known as atherosclerotic plaque that accumulates in blood vessels, leading to the stoppage of oxygen and nutrient supplements to the heart [372, 374]. Since atherosclerosis has been supported to be associated with gut microbiota as described above, the correlation between CAD and gut microbiota has also been researched over the past few years (Fig. 6) [102, 375].

#### The dysbiosis of gut microbiota in CAD

Given that a considerable change in gut microbiota composition has been identified in CAD, microbiota dysbiosis is proposed to play a crucial role in the course of the disease. Studies have shown that some of the gut microbiota are profiled as diagnostic markers in patients with CAD, especially *Clostridium*, *Lactobacillales*, and *Bacteroides*, which are considered diagnostic markers in patients with CAD [376]; *Collinsella aerofaciens* is a possible keystone of CAD triggering different clinical consequences [377]; and *Roseburia*, *Blautia*, and *Ruminococcus* are abundant in CAD patients with in-stent stenosis [378]. In a prospective cohort, both stable CAD and stable CAD combined with T2D patients presented with significantly different metabolite profiles compared to healthy controls [302]. Besides, there was a further shift in metabolite profiles between stable CAD combined with or without T2D, showing that the specific bacteria *Bifidobacterium catenulatum*, *Bifidobacterium longum*, and *Ruminococcus torques* were tightly associated with atherosclerotic severity indicators and adverse cardiac outcomes [302].

#### The metabolites of gut microbiota in CAD

Gut microbial metabolic features have also been proposed as highlights of the various stages of CAD and markers of major adverse cardiac events [102, 375, 379, 380]. For instance, serum choline, carnitine, and TMAO were associated with cumulative events for cardiovascular death in functionally relevant CAD [379]; bacterial co-abundance groups containing operational taxonomic units from *Lachnospiraceae* and *Ruminococcaceae* that produce butyric acid were reduced with progression of CAD [102]. However, the mechanism of gut microbiota and metabolites during CAD development has not been fully elucidated. Hence, revealing and confirming the relationship between gut microbiota and CAD development are essential for introducing novel microbiome-based preventative and therapeutic strategies. Therefore, in this section, we aim to describe the links between microbial-derived metabolism and CAD.

##### TMAO induces inflammation to increase the risk of CAD

TMAO has been mechanistically linked to atherosclerosis and is also proposed as a promotor and predictor of clinical vascular events in CAD [289]. Plasma TMAO levels are associated with long-term risks of cardiovascular events in patients with acute coronary syndrome [381], at least in particular related to its pro-inflammatory capacity, promoting the adhesion ability of aortic endothelial cells. In mechanism, TMAO enhances platelet hyperreactivity by altering Ca^2+^ signaling and elicits a pro-thrombotic effect [359]. Especially, TMAO seems to induce systemic inflammation that contributes to CAD progression. This is particularly relevant given that TMAO directly activates inflammatory pathways, and elicits perturbations in membrane dynamics in human aortic ECs and smooth muscle cells [382, 383]. In particular, the exosomes secreted by TMAO-stimulated hepatocytes increase the secretion of TNF-α and IL-6, and further promote inflammation [383].

##### BAs concentrations change in CAD

Given that BAs, as the main metabolites of cholesterol, exert a powerful effect on maintaining cholesterol homeostasis and anti-atherosclerotic activity, the impaired ability to excrete BAs becomes a risk factor for CAD [384, 385]. Previous studies have found that patients with CAD have lower BAs fecal excretion than CAD-free individuals, indicating the protective effect of BAs excretion against CAD [386, 387]. Notably, recent studies have mentioned that decreased circulating concentrations of BAs are also predictors of CAD [385, 388]. In a sizable cohort study of 7,438 patients with suspected CAD, researchers found that fasting serum total BA levels were highly related to the presence and severity of CAD [388].

Furthermore, it has been uncovered that BAs are associated with regulating channel conductance and calcium dynamics in cardiomyocytes and vascular tone leading to a reduced heart rate [389]. Besides, the molecular action of BAs through binding to TGR5 and FXR drives anti-inflammatory effects in vitro [248, 389]. In this case, cholesterol elimination is involved in the evacuation of intestinal BAs and their sequestrants binding, which leads to lower LDL [248, 389].

##### SCFAs protect the development of CAD

It is notable that gut microbiota could serve as a protective regulator against CAD development by synthesizing SCFAs [390, 391]. In a cohort study with 405 subjects, authors reported the abundance of *Roseburia intestinalis* and *Faecalibacterium cf. prausnitzii*, as the producers of butyrate, was relatively depleted in atherosclerotic CVD, with decreased synthesis of propionate, but no significant changes in acetate [338]. Mechanistically, SCFAs bind to and activate GPR and may exert anti-inflammatory and pro-angiogenic effects in CAD [390, 391].

### The gut microbiota and heart failure

#### The dysbiosis of gut microbiota in heart failure

Indeed, studies mentioned above support that the gut microbiota is tightly associated with the prognosis of atherosclerosis, hypertension, and coronary artery disease, all of which are risk factors for heart failure. Heart failure is one of the leading causes of cardiovascular mortality worldwide and is characterized by higher levels of systemic inflammation [392, 393]. In patients with heart failure, intestinal mucosal edema caused by decreased cardiac output and severe venous blood congestion can bring on the increased bacterial translocation [392], which subsequently aggravates the progression and prognosis of heart failure. Hence, it is no surprise that the increasing number of fecal bacteria corresponds with increased intestinal permeability and underlying systematic inflammation in heart failure [392].

The composition of the gut microbiota in heart failure subjects seems to be altered compared with normal subjects [394]. Heart failure has been linked to specific gut microbial species including *Candida*, *Campylobacter*, *Escherichia coli*, *Klebsiella pneumoniae*, *Shigella*, *Streptococcus viridans*, and *Yersinia* [392, 394]. Toxins of gut microbiota can directly trigger systemic inflammation and have been considered to be critical regulators in the pathological progression of heart failure [392]. As mention above, the main component of the outer membrane of intestinal gram-negative bacteria is LPS, which is an important inflammatory stimulator. LPS can activate TLR4, a membrane receptor that triggers NF-κB signaling, produces pro-inflammatory cytokines, and stimulates the inflammatory response [187, 395, 396]. Patients with heart failure have been shown to have elevated plasma concentrations of LPS during edematous episodes, which may be the key stimuli for cytokine production and immune activation, showing that immune activation in chronic heart failure may be secondary to LPS activation [397].

#### The metabolites of gut microbiota in heart failure

Evidence has declared that the metabolites of gut microbiota exhibit remarkable alterations in heart failure. The gut microbiota, as a metabolic filter, converts nutrients to (microbial-associated) metabolites, including TMAO, SCFAs, BAs, and so on, which affect heart failure progression and prognosis (Fig. 6). Although corroboration of underlying causality between gut microbiota and heart failure is much more complicated and challenging, a better understanding of the mechanisms underpinning microbiota metabolites in development of heart failure could offer new prospects for the primary and secondary prevention.

##### TMAO increases the risk of heart failure

Elevated circulating TMAO levels are linked to myocardial fibrosis in animal models [398] and portend higher long-term mortality risk in patients with heart failure [399–, 400–402]. In a study with a follow-up time of 9.7 years, researchers measured TMAO in 2,490 patients with chronic heart failure, and found that elevated levels of TMAO were predictive of morbidity and mortality in heart failure with reduced ejection fraction (HFrEF) [403]. Besides, it is worth mentioning that the newly published meta-analysis systematically reviewed and provided relatively reliable evidence of the prognostic value of TMAO in heart failure [110]. To date, it is clear that FMO3 could affect the concentration of plasma TMAO by affecting its enzyme activity and consequently improve the prognosis of HFrEF consequently [401]. Besides, in a prospective heart failure cohort, elevated gut microbiota production of N,N,N-trimethyl-5-aminovaleric acid (TMAVA) derived from the TMAO precursor trimethyllysine (TML) was linked with the gradual decrease in fatty acid oxidation and accelerated the risk of cardiac hypertrophy and mortality, but independent of TMAO-mediated metabolic pathways and effects [404].

##### Decreasing the production of SCFAs in heart failure

Since SCFAs as controllers have proven key in maintaining intestinal barrier integrity by regulating ileal motility, mucus secretion, and tight junction protein expression, they may exert beneficial effects on the dysfunctions of intestinal structure and function observed in heart failure [405]. At present, there is little evidence to investigate the direct/indirect relationship between SCFAs and heart failure. In a mouse model of transverse aortic constriction (TAC), the reduction of SCFAs was also observed to be related to the progression and severity of heart failure [406]. In another preclinical study, a high-fiber diet modified the gut microbiota populations and increased the abundance of SCFAs acetate-producing bacteria, resulting in ameliorated cardiac function, by reducing blood pressure, cardiac fibrosis, and hypertrophy [407]. Besides, a remarkable decrease in the abundance of SCFAs-producing bacteria has been found in severe chronic heart failure, presumably leading to the alteration of SCFA pathway-mediated cell function and metabolism of amino acids and carbohydrates [408].

### The gut microbiota and atrial fibrillation

#### The dysbiosis of gut microbiota in atrial fibrillation

Recent studies have suggested that the gut microbiota is associated with another important CVD, atrial fibrillation (AF). AF is one of the most common symptoms of arrhythmias. The incidence of AF in people over 65 years old is about 28‰, and the relatively poor control of AF leads to an increased risk of complications such as heart failure, stroke, and death [409].

Several studies have highlighted the alteration of gut microbiota in patients with AF [410, 411]. Studies have reported an increase in *Rumenococcus, Streptococcus, and Enterococcus,* and a decrease in *Faecali bacterium, Altococcus, Oscillobacter, and Biliophilus* in AF [410]. In patients with persistent AF, an increase in the biodiversity of the gut microbiota and differences in the composition of the microbiota were both observed [410]. The proportions of *Streptococcus* and *Enterococcus* were significantly increased in the gut of patients with AF, while the proportions of bacteria such as *Faecalibacterium* and *Biliophilus* were decreased [410].

In-depth exploration of the mechanism is the key in the prevention and treatment of AF [409]. In recent years, a study has shown that the imbalance of gut microbiota can promote atrial electrical remodeling and structural remodeling by giving rise to intestinal barrier function damage and systemic inflammatory response, which in turn leads to the occurrence and development of AF [412]. Dysbiosis of the aged microbiota leads to impaired glucose tolerance [413]. Moreover, the glucose dysregulation mediated by gut microbiota may be involved in the high incidence of aging-related AF by activating the atrial NLRP3 inflammasome [413].

Studies have suggested that LPS can promote the occurrence of AF by increasing the activity of the NLRP3 inflammasome, and reducing serum LPS may be a potential method for the prevention and treatment of AF [413]. In an animal study, it was found that LPS can increase the expression of pro-inflammatory factors and connexin 43 in cardiomyocytes, increase the expression of L-type Ca^2+^ channel protein, and shorten the effective refractory period of the myocardium, thereby promoting the occurrence of AF [414]. Zhang et al. found that the increase in serum LPS concentration is accompanied by the activation of the atrial NLRP3 inflammasome, which leads to the occurrence of atrial fibrosis [413]. Besides, the selective inhibition of LPS generation and NLRP3 inflammasome activity can inhibit the occurrence of atrial fibrosis [413]. In addition to the structural remodeling, it has been found that upregulation of NLRP3 expression can lead to shortened atrial action potential duration and increased diastolic endoplasmic reticulum Ca^2+^ release frequency, which results in delayed myocardial depolarization and ectopic electrical activity [415].

#### The metabolites of gut microbiota in atrial fibrillation

Several metabolites have been proposed to hint at the link between gut microbiota and AF. In patients with AF, the microbial species that harbored SCFAs synthesis-related genes were decreased [416]. Indoxyl sulfate has an impact on myocardial fibrosis and ventricular remodeling [417]. However, only a few studies have focused on the relationship between metabolites in gut microbiota and AF. Herein, we summarized TMAO and BAs in the development of AF.

##### TMAO increases the risk of AF

It was reported that patients with AF had increased TMAO formation in the gut microbiota [418]. A large cohort study showed that TMAO was positively associated with the incidence of AF [419]. Besides, some studies reported a dose-dependent relationship between circulating TMAO levels in blood and increased AF risk [419, 420], and the risk of thrombus formation [421]. Although the reason why AF patients have higher serum TMAO levels is not completely clear, the mechanism that can explain how TMAO promotes AF has been extensively investigated. TMAO may directly contribute to the development of atrial electrophysiology instability and exacerbate autonomic remodeling in order to induce AF [422]. What’s more, TMAO regulates autonomic nerve conduction through the ganglion plexus, and enhances the activation of the NF-κB-mediated inflammatory signaling pathway so as to induce AF [423]. Nevertheless, one study did not report a direct positive association between TMAO and AF [424], but the precursors of TMAO including choline, betaine, and dimethylglycine played important roles, which were associated with AF risk [424].

##### The different components of BAs modulate the risk of AF

Gut microbiota can affect AF by modulating the metabolic levels of total BAs, and the different components of BAs including DCA and LCA, either by metabolizing bile salts or by modulating downstream signaling pathways of BAs [153]. The dysbiosis of gut microbiota can lead to an increase in the concentration of primary BAs such as CDCA. Previous studies have shown that CDCA can induce cardiomyocyte apoptosis and myocardial fibrosis through the FXR receptor and promote myocardial fibrosis and the occurrence of myocardial damage [425]. Moreover, studies have found that taurocholic acid (TCA) can regulate membrane potential changes in neonatal rat cardiomyocytes, promote the occurrence of AF by stimulating the Na^+^-Ca^2+^ exchanger on the surface of cardiomyocytes, and activate the potassium current regulated by acetylcholine [426]. Abnormal BAs metabolism can lead to lipid metabolism disorders to increase the risk of AF [427]. Therefore, maintaining the metabolic hemostasis of BAs is of great significance for the prevention and treatment of AF.

## Promising therapy of targeting the gut microbiota in CVDs

Previously, we discussed the role of gut microbiota in the development of CVDs, the main mechanism related to the dysbiosis and the concentration change in metabolites. Mounting evidence has emerged in gut microbiota as a novel therapeutic target for the treatment of CVDs. We summarized recent clinical trials (Table 1) and basic research (Table 2) on targeting microbes for the treatment of CVDs. We provided information on several traditional therapeutic strategies including diet interventions, drugs, exercise and surgery, as well as some novel methods such as probiotic and prebiotic therapy and fecal microbiota transplantation (FMT), to identify the efficacy of the above-mentioned therapeutic hotspots in regulating gut microbiota and treating CVDs.

**Table 1:** Clinical trials targeting the gut microbiota in the treatment of cardiovascular diseases

First-author/Year	Models	Intervention	Efficacy	Registration number
**Diet**				
Marilena Vitale 2021 [244]	Overweight/obese individuals	Mediterranean diet	Positive	NCT03071718
Xinyue Zhao 2021 [428]	Patients with CAD	Moderate Alcohol Consumption	Positive	No report
Jalal Moludi 2021 [429]	Patients with CAD	Calorie restriction	Positive	IRCT20121028011288N15
Demir Djekic 2020 [430]	Patients with CAD	Lacto-Ovo-Vegetarian Diet	Positive	NCT02942628
Shyamchand Mayengbam 2019 [431]	Overweight/obese individuals	Dietary fibers	Positive	NCT01719900
Binita Shah 2018 [432]	Patients with CAD	Vegan Diet or the American Heart Association-Recommended Diet	Positive	NCT 02,135,939
Liping Zhao 2018 [246]	Patients with T2D	Dietary fibers	Positive	No report
Luay Rifai 2015 [433]	Patients with heart failure	DASH diet	Positive	No report
Khaider K Sharafedtinov 2013 [434]	Obese hypertensive patients	hypocaloric diet supplemented with probiotic cheese	Positive	ISRCTN76271778
**Probiotics**				
Annefleur Koopen 2022 [435]	Subjects with metabolic syndrome	*A. soehngenii*	Positive	NTR-NL6630
Ayodeji Awoyemi 2021 [436]	Patients with heart failure	*Saccharomyces boulardii*	Negative	NCT02637167
Jalal Moludi 2021 [429]	Patients with CAD	*Lactobacillus rhamnosus GG (LGG)*	Positive	IRCT20121028011288N15
Jalal Moludi 2021 [437]	Patients with MI	*Lactobacillus Rhamnosus G* Inulin	Positive	IRCT20121028011288N15
Jalal Moludi 2019 [438]	Patients with MI	*Lactobacillus rhamnosus capsules*	Positive	IRCT20121028011288N15
Clara Depommier 2019 [439]	Overweight/obese insulin-resistant volunteers	*A. muciniphila*	Positive	NCT02637115
Mobin Malik 2018 [440]	Patients with stable CAD	*Lactobacillus plantarum 299v*	Positive	NCT01952834
Annelise C Costanza 2015 [441]	Patients with heart failure	*Saccharomyces boulardii*	Positive	NCT01500343
Kotaro Aihara 2005 [442]	Subjects with high-normal blood pressure and mild hypertension	*Lactobacillus helveticus*	Positive	No report
**Probiotics and Prebiotics**				
Jalal Moludi 2021 [443]	Patients with CAD	*Lactobacillus Rhamnosus G* and Inulin	Positive	IRCT20180712040438N4
A Hibberd 2019 [444]	Healthy overweight or obese individuals	Polydextrose and *Bifidobacterium animalis subsp*	Positive	NCT01978691
**Prebiotics**				
Christina M van der Beek 2018 [445]	Overweight to obese men	Inulin	Positive	NCT02009670
Alissa C Nicolucci 2017 [446]	Children with overweight or obesity	Oligofructose-enriched inulin	Positive	NCT02125955
Yanan Wang 2016 [447]	Mildly hypercholesterolemic individuals	β-glucan	Positive	NCT01408719
Evelyne M Dewulf 2013 [448]	Obese women	Inulin-type fructans©	Positive	NCT00616057
**Exercise**				
Joanna Cwikiel 2017 [449]	Patients with CAD	Bicycle ergometer	Negative	NCT01495091
Susanne Kristine Aune [450]	Patients with CAD	Exercise stress testing	Negative	NCT01495091
Elizabeth A Rettedal 2020 [451]	Overweight participants	High-intensity interval training	Positive	ACTRN12617000472370
**Drug**				
Yifei Zhang 2020 [452]	Patients with T2D	Berberine and probiotics	Positive	NCT02861261
W H Wilson Tang 2013 [453]	Patients undergoing elective coronary angiography	broad-spectrum antibiotics	Positive	No report
Adam F M Stone 2002 [454]	Patients admitted with acute MI or unstable angina	Amoxicillin, metronidazole	Positive	No report
**FMT**				
Hao-Jie Zhong 2021 [455]	Hypertensive patients	Washed microbiota transplantation	Positive	No report
Jessica R Allegretti 2020 [456]	Obese patients	FMT capsules	Positive	NCT02741518
Loek P Smits 2018 [457]	Patients with metabolic syndrome	Vegan FMT	Positive	NTR 4338
Anne Vrieze 2012 [458]	Patients with metabolic syndrome	FMT	Positive	NTR1776

*Abbreviation*: *CAD* Coronary artery disease, *MI* Myocardial infarction, *T2D* type 2 diabetes

**Table 2:** Basic researches of targeting gut microbiota in the treatment of cardiovascular diseases

First-author/Year	Diseases	Intervention	Efficacy
**Diet**			
Deyang Yu 2021 [459]	Obese mice	Isoleucine or Valine	Negative
Tulika Arora 2019 [460]	High-fat-fed mice	Dietary fibers	Positive
Nicole E Cummings 2018 [280]	Diet-induced obese mice	Specifically reducing dietary BCAAs	Positive
**Probiotics**			
Ryan du Preez 2021 [461]	Rats fed either corn starch or high-carbohydrate, high-fat diets	*N. oceanica*	Positive
Victoria L O'Morain 2021 [371]	High-fat-fed LDLR -/- mice	Lab4P	Positive
Naofumi Yoshida 2021 [223]	Diet-induced obesity mice	*Bacteroides spp.*	Positive
Iñaki Robles-Vera 2020 [462]	Spontaneously hypertensive rats	BFM and *lactobacillus fermentum* cect5716 (lc40)	Positive
Iñaki Robles-Vera 2020 [463]	Hypertension in deoxycorticosterone acetate (DOCA)-salt rats	BFM	Positive
Mona Mischke 2018 [464]	Mice with a high-fat Western-style diet	Engineered probiotic bacteria and specific symbionts	Positive
Eunjung Lee 2018 [465]	White adipose tissues of mice	*Lactobacillus plantarum strain* Ln4	Positive
Lingling Jia 2017 [466]	Diabetic mice induced by high fat diet plus streptozotocin	*Clostridium butyricum*	Positive
D R Michael 2017 [467]	High-fat-fed mice	Lab4P	Positive
Zhen-Lin Liao 2016 [468]	High-fat-fed ApoE -/- mice	*Bifidobacteria*	Positive
Xiaohong Tracey Gan 2014 [469]	Post-MI rats	*Lactobacillus rhamnosus GR-1*	Positive
Qifang Wu 2021 [470]	T2D mice	Sargassum fusiforme fucoidan	Positive
Qichao Chen 2019 [471]	High-fat diet-induced dyslipidemia in rats	Fucoidan and galactooligosaccharides	Positive
**Prebiotics**			
Emilie Catry 2018 [472]	ApoE -/- mice fed an n-3 polyunsaturated fatty acid-depleted diet	Inulin-type fructans	Positive
Lisa R Hoving 2018 [473]	Atherosclerosis in hypercholesterolemic APOE*3-Leiden.CETP mice	Inulin	Negative
Lourdes Fernández de Cossío 2017 [474]	Mice with metabolic syndrome	Oligofructose	Positive
P D Cani 2007 [475]	High-fat-fed mice	Oligofructose	Positive
Marie-Hélène Rault-Nania 2006 [476]	ApoE -/- mice	Inulin	Positive
**Drug (Antibiotics)**			
Xuefang Yan 2020 [477]	Rats with high-salt diet	Quadruple antibiotic treatment	Positive
S Galla 2018 [478]	Spontaneously hypertensive rats	Minocycline and vancomycin	Positive
S Galla 2018 [478]	Salt-sensitive rats	Minocycline and vancomycin	Negative
Vy Lam 2016 [380]	Post-MI rats	Vancomycin	Positive
**Drug (TCM)**			
Song Yang 2021 [479]	High-fat-fed mice	Akebia saponin D	Positive
Boran Zhu 2020 [480]	High-fat-fed mice	Alisma orientalis beverage	Positive
Jianbo Wu 2020 [481]	WKY/Izm rats	Baicalin and berberine in Sanoshashinto	Positive
Zhiyong Du 2020 [482]	Cardiac hypertrophy rats	Baoyuan decoction	Positive
Patricia Diez-Echave 2020 [483]	High-fat-fed mice	Lippia Citriodora Extract	Positive
Dandan Wu 2019 [484]	Spontaneously hypertensive rats	Baicalin	Positive
Xiaoying Yu 2019 [485]	Spontaneously hypertensive rats	Zhengganxifeng Decoction	Positive
Yuqing Meng 2018 [486]	Post-MI rats	Notoginseng	Positive
Ming-liang Chen 2016 [487]	TMAO-induced atherosclerosis in ApoE(-/-) mice	Resveratrol	Positive
Jing-Hua Wang 2014 [488]	High-fat-fed rats	Flos Lonicera	Positive
Zhen-Li Wu 2014 [489]	Post-MI rats	Sclederma of Poria cocos (Hoelen)	Positive
**Drug (Microbial TMA-lyase inhibitors)**			
Chelsea L Organ 2020 [490]	Murine model of heart failure	Diet containing choline plus a microbial choline TMA-lyase inhibitor	Positive
Kui Chen 2017 [398]	Mice with a Western diet	DMB	Positive
Zeneng Wang 2015 [491]	Mice fed a high-choline or L-carnitine diet	DMB	Positive
**Exercise**			
Wen-Jie Xia 2021 [492]	Spontaneously hypertensive rat	Moderate-intensity exercise	Positive
Zuheng Liu 2017[493]	Post-MI mice	Moderate-Intensity Exercise	Positive
Bernardo A Petriz 2014 [494]	Obese, non-obese and spontaneously hypertensive rats	Controlled exercise training	Positive
**FMT**			
Yun Zhang 2022 [413]	Aged rats	FMT	Positive
Eun Sil Kim 2022 [495]	Atherosclerosis-prone mouse model	FMT	Positive
Xuefang Yan 2020 [477]	High salt-induced hypertension rats	FMT	Positive
Marta Toral 2019 [496]	Spontaneously hypertensive rats	FMT	Positive

*Abbreviation*: *CAD* Coronary artery disease, *MI* Myocardial infarction, *T2D* Type 2 diabetes, *LDLR* Low-density lipoprotein receptor, *Lab4P* Lab4 probiotic consortium (*bifidobacterium bifidum*, *bifidobacterium animalis* subsp. *Lactis* and two strains of *lactobacillus acidophilus* plus *lactobacillus plantarum* cul66, *BFM Bifidobacterium breve* cect7263, *BCAAs* Branched-chain amino acids, *DMB* 3,3-Dimethyl-1-butanol, an inhibitor of trimethylamine formation, *FMT* Fecal microbiota transplantation

### Diet

Importantly, adding SCFAs or using diet enriched SCFAs could stimulate the expression of leptin and decrease food intake as well as energy storage in order to treat obesity [497]. Intervention with a traditional Mediterranean diet could increase the abundance of Intestinimonas butyriciproducens and A. muciniphila, and increase postprandial plasma butyric levels [244]. Avocado treatment could also increase Faecalibacterium, Lachnospira, and Alistipes, increase fecal SCFAs concentrations and decrease BAs concentration [498]. Besides, supplementing fiber content in obese mice can significantly increase energy expenditure and the number of beneficial bacteria, and it also inhibits weight gain through the SCFAs-GRP41 pathway [460].

Furthermore, modulation of BAs by diet may be a promising treatment for CVDs risk factors [499]. Polyphenol- or polyphenol-rich foods increase the metabolic output of BAs and beneficial bacteria such as Bifidobacterium, Lactobacillus, Akkermansia, Bacteroides, and Eubacterium [500]. In addition, dietary fiber supplementation could also increase fecal acetate and reduce fecal cholate, deoxycholate and total BAs contents to cause weight loss [431]. Additionally, regulating the protein in the diet shows great benefit and a protein-deprived diet can reverse obesity in mouse models [501]. Specifically, dietary intervention is the key method for the adjustment of BCAAs. Studies have shown that interventions based on reducing dietary levels of BCAAs are a new approach for the treatment and prevention of obesity and diabetes [226]. Reducing dietary BCAAs rapidly reverses diet-induced obesity and improves glycemic control in obese mice, suggesting that reducing dietary BCAAs may be helpful in the treatment of obese and insulin-resistant patients. However, this finding needs to be further verified in the human population [280].

Of course, diet is very important in the treatment of diabetes. Increasing the intake of dietary fiber can upregulate the synthesis of SCFAs, which promotes the secretion of insulin and insulin sensitivity, and relieves the symptoms of T2D in turn [246]. A special diet with butyric acid may improve glucose metabolism and insulin sensitivity [244]. Moreover, regulating meal timing and dietary macronutrients may also regulate gut microbiota and the metabolism of glucose [502, 503]. Targeting BCAAs is also a promising method to treat diabetes. By adding tryptophan or threonine to balance the ratio of amino acids in the diet, excess food intake can be suppressed [119]. On the other hand, the reduction of isoleucine and valine can significantly improve glucose tolerance, but the reduction of leucine does not improve glucose tolerance in mice, because of the regulation of the FOXA2-FGF21 signaling pathway [459, 504]. BCAAs restriction by using an amylopectin diet restricted weight loss and improved blood glucose levels in mice [280]. In humans, BCAAs restriction has also been found to reduce systemic BCAAs levels, enhance glucose sensitivity, and reduce postprandial insulin levels [280].

There are some studies explored the treatment of CVDs by diet [505, 506]. Dietary regulation can affect the changes in the composition of intestinal microorganisms and significantly affect the various metabolites produced by gut microbiota [507]. For example, it has been reported that the metabolism of dietary L-carnitine by intestinal microorganisms also produces TMAO, which leads to atherosclerosis [160]. The Mediterranean diet can effectively promote the proliferation of SCFAs-producing bacteria in the intestinal tract, reduce chronic inflammation, and thus stabilize blood pressure [508]. Additionally, a Mediterranean diet was reported to reduce AF risk, while low-carbohydrate diets may increase the risk of AF [509, 510]. It was found that a low-calorie diet combined with probiotics helps to reduce BMI and blood pressure in obese patients with hypertension [434]. In addition, other dietary patterns, such as dietary approaches to stop hypertension (DASH) diet, potassium-rich substitute, and intermittent fasting, can also regulate the structure of gut microbiota and SCFAs production, which help to regulate blood pressure [316]. Dietary fiber is thought to effectively reduce the risk of CVDs by lowering LDL levels in blood plasma [511]. Low dietary fiber intake will not only lead to a decrease in microbial diversity and SCFAs production, but also affect nitrogen balance [512].

Since dietary interventions may play a role in secondary cardiovascular prevention, recent clinical studies have explored the effects of diet on attenuating cardiovascular events in CAD [429, 430, 432, 513]. Calorie restriction [429], the Vegan diet [432, 513], the American Heart Association–recommended diet [432, 513], and the Lacto-Ovo-Vegetarian diet [430] showed the improvements in cardiometabolic risk factors by regulating levels of serum high-sensitivity C-reactive protein and lipotoxic lipids, as well as plasma and urinary levels of TMAO. In addition, although it is not yet possible to draw conclusions that moderate alcohol drinking is linked to a lower risk of adverse cardiovascular events [514], a study noted that moderate consumption tended to have more positive effects on metabolic profiles and commensal microbiota in 72 patients with CAD [428].

With strong associations in heart failure, we discussed and looked ahead dietary interventions for targeted regulation of gut microbiota metabolites. Choline and TMAO diets could exacerbate cardiac dilatation and left ventricular dysfunction, leading to aggravated heart failure [515]. Multiple observational studies suggest that the DASH diet, which is abundant with fruits, vegetables, whole grains, and low-fat dairy products could decrease heart failure incidence and severity [516–519]. For example, in a previous randomized controlled trial, subjects with heart failure assigned to the DASH group followed the DASH eating plan for 3 months and demonstrated better exercise capacity and quality of life [433]. Furthermore, the fasting-mimicking diet (FMD), a caloric restriction dietary intervention has been applied in a clinical study, indicating the benefit of FMD in reducing plasma levels of TMAO and the risk of CVDs [520].

### Probiotic and prebiotic therapy

#### Probiotics

Probiotics are a general term for microorganisms that can improve the microecological balance of the host and play a beneficial role in the intestinal tract [521]. Regulating the gut microbiota using probiotics may be a good supplementary treatment for obesity and the regulation of lipid metabolism [522–524]. It was reported that *Bacteroides* could be used for treating obesity [223]. Nevertheless, whether adding probiotic strains has an effect on the adjustment of the composition of intestinal microbiota, insulin resistance and inflammation remains to be investigated [525]. Some studies have used bacteria to promote BCAAs metabolism to explore potential bacterial therapeutic effects. For instance, *Bacteroides spp.* was used to treat obesity by promoting the breakdown of BCAAs [223]. Moreover, engineered probiotic bacteria and specific symbionts have shown a beneficial effect on reducing obesity in mice, although the relative methods that affect obesity risk have yet to be determined [464].

Some specific kinds of probiotics can also regulate the occurrence, development and treatment of diabetes. One of the most intensively investigated probiotics is *A. muciniphila* supplementation. *A. muciniphila* abundance, one of the main probiotics, is associated with ameliorating insulin sensitivity and inflammation [526]. A decreased amount of *A. muciniphila* is associated with the occurrence and development of T2D [200]. Administration of *A. muciniphila* to mice fed with a high-fat diet reversed their increased fat mass, alleviated metabolic endotoxemia, improved adipose tissue inflammation and insulin resistance, and decreased inflammation-related markers [527]. Likewise, supplementation with *A. muciniphila* could improve metabolic health in humans [528].

Other kinds of probiotics may also play a beneficial role. Studies have suggested that supplementation with *Clostridium butyricum* in diabetic mice could reduce systemic insulin resistance and inflammation, increase mitochondrial metabolism, and significantly reduce gut damage [466]. Besides, *N. oceanica* has applicability to produce biofuels and it was also reported to improve the abundance of *Oxyphotobacteria* in order to treat metabolic syndrome [461]. *Lactobacillus plantarum strain* Ln4 administration changes the expression of several hepatic genes (increased mRNA levels of IRS2, Akt2, and AMPK and reduced CD36) that regulate glucose and lipid metabolism [465]. Treatment with *A. soehngenii* may cause the release of GLP-1 and induce the expression of Regenerating Family Member 1 Beta, thereby regulating blood glucose [435].

It is important that probiotics can regulate lipid metabolism, produce SCFAs, regulate TMAO levels, and play a regulatory role in lowering the blood pressure [333, 462]. It has been reported that the probiotics *Bifidobacterium breve* CECT7263 (BFM) and *Lactobacillus fermentum* CECT5716 (LC40) could prevent endothelial dysfunction and high blood pressure in inherited hypertension [462]. Another study found that BFM could reduce blood pressure in renin-independent hypertensive rats, and its mechanism might be related to the decrease in plasma trimethylamine [463]. A previous clinical study suggested that daily powdered fermented milk with *Lactobacillus helveticus* can lower blood pressure in patients with hypertension without any adverse reactions [442]. In fact, a number of meta-analyses have shown a decrease in systolic and diastolic blood pressure in hypertensive patients after taking probiotics [529–531].

Many studies have fully investigated the role of probiotics in the prevention and treatment of atherosclerosis [532–534]. For example, *Lactobacillus* can regulate gut microbiota structure and its metabolites, and improve lipid metabolism and serum TMAO levels [535]. It was found that dietary tea polyphenols could affect intestinal *Bifidobacteria* and regulate atherosclerotic plaques [468]. P Hlivak et al. studied the effects of long-term oral administration of *Enterococcus faecium* M-74 rich in selenium on human total cholesterol, LDH, HDL and triglycerides, and they found that the application of *Enterococcus faecium* M-74 probiotics for 1 year can reduce serum cholesterol levels [536]. In addition, the Lab4 probiotic consortium (*Bifidobacterium bifidum*, *Bifidobacterium animalis* subsp. *lactis* and two strains of *Lactobacillus acidophilus*) plus *Lactobacillus plantarum* CUL66 (Lab4P) can reduce plasma cholesterol levels induced by a high-fat diet [467]. Researchers further demonstrated that Lab4P reduced atherosclerosis in LDLR^−/−^ mice and stabilized plaques by inhibiting inflammation [371].

Of note, there are increasing numbers of clinical trials goaled to evaluate the effects of probiotic administration in patients with CAD or myocardial infarction [429, 437, 438, 440, 443]. Probiotics can restore gut barrier integrity and lessen intestinal permeability, causing a drop in LPS levels [429, 537]. After 12 weeks of intervention with *Lactobacillus rhamnosus GG*, the serum levels of IL-1β and LPS decreased, showing promising effects on inflammation and metabolic endotoxemia in CAD patients [429]. Furthermore, in a pilot study of 21 men with stable CAD, supplementation with *Lactobacillus plantarum 299v* rescued dysfunctional vascular endothelial cells, and decreased systemic inflammation, independent of changes in traditional CVDs risk factors and TMAO [440].

As mentioned above, the nature of the bacterial flora in patients with heart failure differed from that in control subjects, which implies the potential therapeutic effect of targeting and shifting the composition of gut bacteria in heart failure. From this perspective, selective administration of probiotics, which have cardioprotective benefits, might be deemed a potential strategy for primary and secondary prevention of heart failure [394, 538]. Gan et al. provided evidence that oral *Lactobacillus rhamnosus* GR-1 oral administration can attenuate the progression of heart failure, and maintain an anti-remodeling effect after withdrawal [469]. Additionally, daily oral administration with *Saccharomyces boulardii* showed benefits for chronic systolic heart failure, presenting as elevated left ventricular ejection fraction (LVEF), along with decreased circulating inflammatory mediators [441]. These observations pointed that the use of probiotics could provide additional benefits in heart failure treatment, specifically reducing the severity and improving the prognosis of heart failure post-myocardial infarction. However, there was a multicenter study with inconsistent conclusions that treatment with *Saccharomyces boulardii* or *rifaximin* had no significant effect on LVEF and microbiota diversity in patients with heart failure [436].

#### Prebiotics

Prebiotics are typically molecules that are difficult to digest in foods, such as oligosaccharides or complex sugars. As food additives, they can improve host health by selectively stimulating gut bacteria, including probiotics [539, 540]. For example, prebiotics also have beneficial effects on obesity by improving glycemic control and plasma lipid profiles [475, 511]. Supplementation with fructo-oligosaccharides and biotin limits weight gain and glycemic deterioration in high-fat diet fed mice [541]. However, some studies proposed that treating obesity patients with prebiotics did not show a strong effect on weight loss, and the reason may be due to the individualized bacterial diversity [448, 542].

Additionally, inulin-type fructans promote the abundance of NO-producing bacteria, and decrease secondary BAs-producing bacteria [472]. Fiber, polyphenols, and certain drugs can sequester BAs, reducing their entry into the colon and the metabolism of colon bacteria [543]. Treatment of decreasing circulating BAs and increasing fecal BAs using prebiotics shows some benefits. Ileo-colonic delivery of conjugated BAs improved glucose homeostasis and increased fecal BAs [544]. In children with obesity, primary BAs increased in the control group but not in the oligofructose-enriched inulin treated groups [446]. Several types of plasma BAs were reduced in synbiotic treatment combining a prebiotic (polydextrose) and probiotic (*Bifidobacterium animalis subsp*, B420) compared to placebo in overweight adults [444].

Administration of prebiotics may also play a role by reversing the specific signatures of gut microbiota associated with diabetes, thereby improving intestinal permeability, controlling endotoxemia, reducing inflammation, and eventually improving glucose tolerance [474]. Some kinds of prebiotics, such as Sargassum fusiforme fucoidan, were reported to be an adjuvant agent to treat T2D well [470]. Fructose oligosaccharides can improve glucose metabolism and insulin sensitivity in patients with T2D [545]. Moreover, oral administration of prebiotics, such as galacto-oligosaccharides and resveratrol, can modulate the structure of gut microbiota and reduce the levels of TMAO and LPS in circulation in rats with high-fat diet-induced dyslipidemia [471].

Moreover, it was found that beta-glucan alters the composition of gut microbes and lowers blood pressure [447, 546]. Previous studies have shown that inulin can reduce atherosclerosis in ApoE deficient mice [476]. Besides, prebiotic inulin improves fat oxidation and promotes SCFAs production in overweight or obese men, which is beneficial for substrate metabolism and may have a positive effect on the improvement of atherosclerotic symptoms [445]. Emilie Catry et al. investigated the effect of gut microbiota on vascular dysfunction in ApoE^−/−^ mice. Supplementation with inulin-type fructans could activate the NOS pathway and completely reverse mesentery and carotid endothelial dysfunction in mice [472]. Oppositely, it has also been reported that inulin can regulate gut microbiota, but cannot improve atherosclerosis in hypercholesterolemic ApoE*3-Leiden. CETP mice [473].

Interestingly, in a randomized, double-blind clinical trial, the co-supplementation of inulin as a prebiotic with probiotic *Lactobacillus rhamnosus*, was shown to have beneficial effects on depression, anxiety, and the level of microbial translocation in patients with CAD [443]. In summary, diet may be a promising therapy for the treatment of CVDs by targeting gut microbiota.

### Drug

#### Antibiotics

The use of antibiotics can reduce harmful microbiota, regulate the metabolites of gut microbiota, and improve the prognosis of CVDs [547]. Many antibiotics have been reported to affect gut microbiota and blood pressure. For instance, it has been reported that minocycline can change the composition of gut microbiota and regulate blood pressure in hypertensive rats [548]. And elevated systolic and diastolic blood pressure in salt-induced hypertensive rats decreased significantly after quadruple antibiotic treatment [477]. Although it seems that antibiotics can lower blood pressure by changing gut microbiota, some studies have come to different results [549]. For example, minocycline and vancomycin reduced the abundance of *Firmicutes* in the intestinal tract of rats, which decreased blood pressure in spontaneously hypertensive rats, but led to an increase in blood pressure in salt-sensitive rats [478].

Excitedly, it has been found that antibiotics can effectively reduce the levels of TMAO in plasma [338, 453]. Ciprofloxacin and metronidazole can reduce the microbiota that can convert choline into TMA, so as to inhibit the formation of macrophage foam cells and improve the progression of atherosclerosis [550]. However, antibiotics affect the diversity of gut microbiota, and long-term use has been identified as an independent risk factor for atherosclerotic events [551]. It has been shown that the increase in atherosclerosis caused by antibiotics is related to the loss of intestinal diversity and the change in microbial metabolic function, which limits the use of antibiotics as a practical treatment of anti-atherosclerosis [551].

In addition, antibiotics are currently used for gut microbiome-based CAD treatment. Antibiotic treatment has primarily focused on the elimination of disease-causing microbiota to alter the progression of CAD. A previous study found that the broad-spectrum antibiotic vancomycin altered the abundance of gut microbiota and levels of circulating leptin, showing benefits of infarct area reduction and cardiac function recovery post-ischemia [380]. However, the effect of antibiotic treatment on reducing adverse cardiac events in patients with CAD is still conflicting [454, 552]. It has been claimed that antibiotics macrolides and quinolones show harmful effects on cardiac prognosis for the secondary prevention of CAD in the clinic [454, 552]. In general, more clinical studies with a longer follow-up duration that assess the safety of antibiotics on CAD are needed.

There is evidence that antibiotics may play a role in CVDs, but the possible drug resistance caused by long-term use of antibiotics is also of concern, as long-term use of antibiotics may disrupt the dynamic balance of intestinal microorganisms [553]. Therefore, based on the current findings, we believe that more preclinical and clinical studies are needed to clarify the role of antibiotics in the gut microbiota and CVDs, and individualized use of antibiotics in patients with CVDs should also be considered.

#### Traditional Chinese medicine

Traditional Chinese medicine (TCM) can protect intestinal barrier function and regulate the biosynthesis of SCFAs, BAs, and tryptophan to treat metabolic diseases [554]. Lippia citriodora lessens intestinal dysbiosis by reducing the *Firmicutes* to *Bacteroidetes* ratio and increasing the abundance of *A. muciniphila* to reduce fat accumulation and improve plasma glycemic control and lipid profiles [483]. Akebia saponin D was able to downregulate the PPAR-γ pathway in order to decrease plasma lipids and insulin resistance [479]. Other TCMs, such as berberine, have also been shown to lower blood glucose by regulating the effects of gut microbiota on BAs metabolism [452].

Additionally, TCM may be a promising treatment for CVDs. Recent studies have shown that TCM may improve the symptoms of hypertension by regulating gut microbiota [555]. Baicalin and berberine have been found to reduce left ventricular hypertrophy and change the intestinal microbiota to improve hypertension [481]. Besides, baicalin can regulate the permeability of the intestinal epithelial barrier, reduce inflammatory factors and reduce the inflammatory response in spontaneously hypertensive rats [484]. In addition, TCM prescriptions Zhen Gan Xi Feng Decoction and Sanoshashinto (San Huang Xie Xin Tang in Chinese) have also been found to regulate the diversity of gut microbiota and their metabolites to reduce blood pressure [481, 485].

An increasing number of studies have shown that the active components of TCM can regulate the structure of gut microbiota, reduce inflammatory reactions, and improve atherosclerosis [556–558]. Besides, Ganoderma lucidum, Honeysuckle and other TCMs have also been found to effectively delay the development of atherosclerosis [487, 488]. The Alisma orientalis beverage is a TCM prepared by a variety of medicinal plants that can be used for atherosclerosis. Its mechanism may be the change in gut microbiota content and the decrease in gut microbiota metabolite TMAO [480].

A growing body of evidence has mentioned that gut microbiota, which emerged as the frontier, may play an important role in the mechanism underlying TCM in heart failure [559–561]. Du et al. demonstrated that *Baoyuan Decoction* formula could regulate gut metabolism-related tryptophan and attenuate cardiac hypertrophy, which contributes to alleviating the development of heart failure [482]. Besides, other TCMs, such as Notoginseng total saponins, Safflower total flavonoids [486], and Poria cocos [489], can dilate blood vessels, and diuresis and inhibit adverse cardiac remodeling.

#### Microbial TMA-lyase inhibitors

Of note, the association between TMAO levels and clinical adverse consequences in CVDs has been shown in clinical studies [562]. Besides, the structural analog of choline, 3,3-dimethyl-1-butanol, could reduce the level of TMAO via restraining the activity of choline TMA-lyase and alleviate cardiac function and fibrosis [398]. In fact, microbial TMA-lyase inhibitors focused on the inhibition of TMAO production may alter the progression of heart failure. It has been claimed that dietary withdrawal of TMAO as well as administration of a gut microbe-targeted inhibitor of TMAO production, exert promising effects on heart structure and function during heart failure in a murine model [490].

Furthermore, one study used 3, 3‐dimethyl‐1‐butanol to suppress microbial TMA and TMAO formation in the treatment of atherosclerosis [491]. They found that 3, 3‐dimethyl‐1‐butanol inhibited the generation of TMAO, reduced the formation of macrophage foam cells, and decreased the development of atherosclerosis [491]. However, considering the crucial role of TMAO in the development of atherosclerotic plaques, whether inhibiting the TMAO pathway can prevent the progression and improve the prognosis of other types of CVDs remains to be determined. In short, microbial TMA-lyase inhibitors have certain clinical transformation potential in the treatment of CVDs.

#### Other drugs

Some drugs of cardiovascular systems such as statin, glucose-lowering drugs, and blood pressure lowering drugs are reported to regulate gut microbiota [563–, 564–566]. Researchers found that some inhibitors, including simvastatin and atorvastatin could regulate gut microbiota in the prevention and treatment of hyperlipidemia [563, 564]. Statin treatment could also decrease the degree of obesity-associated microbiota dysbiosis, characterized by a relatively higher proportion of *Bacteroides* and a lower proportion of *Faecalibacterium* after inhibitor treatment [567]. Anti-diabetes drug interventions may have a significant impact on the microbiota composition and function, independent of their effects on glycemic control [568, 569]. These drugs may regulate the abundance of SCFA-generating bacteria and the *Firmicutes* to *Bacteroidetes* ratio [570, 571]. For example, acarbose, and sitagliptin could increase the number of beneficial bacteria in the gut while lowering blood glucose [572]. In addition, metformin increased the levels of SCFAs including butyrate, acetate, and valerate, and altered three glucose metabolism pathways (glycolysis, aerobic oxidation, and pentose phosphate) compared with controls [573]. Metformin also increased *A. muciniphila* to regulate glucose homeostasis [574, 575]. Furthermore, some studies have found that drugs such as vitamin D and metformin can also reduce TMAO levels by affecting the composition and function of gut microbiota [566, 576, 577], thus treating atherosclerosis. Moreover, drugs targeting the BAs receptor FXR also have promising effects on non-alcoholic fatty liver disease [578, 579], which is known to be clinically associated with CVDs. However, to date, the ability of BAs sequestrants to decelerate CAD progression remains ambiguous, and BAs receptor-targeting drugs have been demonstrated in CAD treatment.

### Exercise

Previous studies have suggested that exercise can change the composition and diversity of gut microbiota in hypertensive rats [494]. Exercise can continuously decrease systolic blood pressure in spontaneously hypertensive rats through the remodeling of gut microbiota [492]. In addition to basic research, several clinical trials have explored the role of exercise in regulating gut microbiota in hypertensive patients. Previous clinical trials found that the imbalance of gut microbiota may be a potential cause of decreased exercise ability in elderly patients with hypertension [580], and exercise can effectively regulate the richness of gut microbiota and its metabolites, which is beneficial to the control of hypertension [581].

Exercise training has been known as an evidence-based therapeutic intervention for CAD [582]. The decreased morbidity and mortality in CAD patients benefit from increasing coronary blood flow and myocardial oxygen demand [583]. Exercise might be a promising tool to alter the gut microbiota in CAD, given that regular exercise could play a beneficial role in gut health, including preventing gut dysbiosis, increasing microbial diversity, and decreasing circulating levels of gut leakage markers both in murine models and humans [493, 584, 585]. In a mouse MI model, it was confirmed that moderate-intensity exercise could alter the gut microbial composition and improve cardiac function [493]. However, notably, strenuous exercise triggers myocardial infarction through elevated pro-coagulant activity and levels of gut leakage markers [449, 450]. Furthermore, strenuous exercise is associated with a higher level of gut leakage, which significantly increases LPS, LPS-binding protein, and soluble CD14 levels in patients with symptoms of CAD [450]**.** All of the above results indicated the essentiality of exercise intensity in CAD treatment.

Oppositely, there is little evidence about the effect of exercise on regulating gut microbiota in the treatment of obesity. Although exercise could not regulate the diversity or community structure of gut microbiota, it could improve the abundance of certain microbiome genera including *Coprococcus, Blautia, Lachnospiraceae* and *Dorea* to increase insulin sensitivity in patients with obesity [451]. In brief, the regulation of gut microbiota composition and its metabolites via exercise may be a promising therapy.

### Surgery

Bariatric surgery has been reported to alter the microbiota and increase circulating levels of primary and secondary BAs [586–588]. Besides, it is currently known that bariatric surgery, such as gastric bypass surgery and sleeve gastrectomy, affects BAs metabolism to regulate fat metabolism, and then achieve weight loss [589]. Additionally, bariatric surgery has been reported to upregulate FXR signaling in order to exert its function [590, 591]. And one study has suggested that in patients receiving vertical sleeve gastrectomy, changes in gut microbiota are important factors in weight loss and blood glucose control [590]. After Roux-e-Y gastric bypass surgery or vertical sleeve gastrectomy, the effect of improving insulin resistance and abnormal glucose tolerance is obvious, accompanied by a change in the diversity of the microbiota and an increase in the plasma total BAs levels [592, 593]. Taken together, surgery may provide a new perspective for regulating intestinal flora in obese or diabetic patients and a new idea for reducing cardiovascular disease risk factors.

### Fecal microbiota transplantation

Fecal microbiota transplantation (FMT) aims to treat CVDs by replacing intestinal pathogens by introducing fecal contents from healthy subjects into the patient's gastrointestinal tract. FMT treatment for patients with obesity led to sustained changes in the intestinal microbiome, increased butyrate-producing bacteria, readjustment of glucose homeostasis and BAs profiles [456, 458]. A study found that single transplantation of fecal microbiota from leptin donors in patients with metabolic syndrome could lead to changes in the composition of gut microbiota [457]. Another study found that FMT resulted in increased insulin sensitivity, weight loss, and increased microbial diversity [458].

Excitedly, previous studies have found that hypertension can be controlled by transplanting gut microbiota from normal rats into the intestinal tract of high-salt-induced hypertensive rats [477]. In fact, FMT improves blood pressure symptoms by increasing the number of SCFAs-producing bacteria and improving intestinal permeability [291]. In addition, it has been reported that FMT can lower blood pressure by reducing the production of pro-inflammatory cytokines [496]. A recent retrospective study found that both systolic and diastolic blood pressure decreased in hypertensive patients after transplantation of detergent bacteria from normotensive donors to hypertensive patients [455].

Moreover, FMT can increase the abundance of butyric-producing bacteria, suggesting that FMT might be a potential treatment strategy for atherosclerosis [458]. More importantly, new research has found that FMT can improve atherosclerosis in C1q/TNF-related protein 9 gene deficient mice [495]. In addition, FMT can improve atrial fibrosis and reduce the expression level of NLRP3, thereby reducing susceptibility to AF [413].

However, the use of FMT is currently severely restricted due to the adverse effects associated with FMT, including the potential transfer of LPS and infectious agents [594, 595]. It has also been suggested that transplantation of specific species of bacteria in the gut microbiota may be a reasonable alternative to FMT [596]. More studies are needed to explore whether FMT is an effective and safe therapeutic method for CVDs and their risk factors. Future research should focus on the mechanistic interaction between these therapies and host metabolism.

## Perspective

The gut microbiota interacts with the host to form a mutualistic environment for both microbiota and health, which regulates host intestinal barrier function, the immune system as well as material metabolism. Conversely, dysbiosis of gut microbiota disrupts normal function, induces inflammation, and releases harmful metabolites to trigger diseases. Excitedly, the relationship between gut microbiota and diseases suggests that the gut microbiota may be a potential novel therapeutic target. Therefore, identifying the underlying mechanistic relationship linking gut microbiota and diseases would do a great favor to prevent the progression of diseases.

The gut microbiota in the human body is affected by a variety of external factors, among which food and drugs play important roles. A healthy diet can not only maintain a normal energy balance, but also regulate the composition and diversity of the human gut microbiota, thus affecting the occurrence and development of diseases [597]. Take CVDs for example, the Mediterranean diet has many benefits, such as lowering blood cholesterol and the level of inflammatory factors [505]. In addition, increasing the dietary fiber content or a calorie-restricted diet, and exercise training can also modulate energy balance, thereby promoting health and reducing the incidence of disease [429, 598]. In fact, CVDs are thought to be related to excess energy, disorder of substance metabolism, and insulin resistance [599]. Previous studies have shown that the gut microbiota is also associated with energy intake, material metabolism, and insulin sensitivity [526, 600]. Therefore, are there more “obese bacteria” that promote the development of CVDs by increasing energy intake from food in patients with CVDs than in healthy people [601]? Does excessive calorie intake and lack of physical exercise promote the development of CVDs by reshaping gut microbiota [492]? Future research that clarifies the role of gut microbiota in health and disease could be helpful for us to address the above-mentioned questions. Additionally, exploring the role of gut microbiota in health and CVDs will help to develop novel therapeutic interventions, including increasing vegetarian and dietary fiber content, restricting caloric intake, using probiotics/prebiotics or FMT regulation, and increasing exercise training, to prevent gut microbiota dysbiosis and reduce the occurrence of CVDs.

Currently, established drugs can regulate the gut microbiota, which in turn blocks or reverses the progression of CVDs. Metabolites produced by gut microbiota may be pharmacological targets for treatment. Exploring the causative effects of metabolites in disease susceptibility may provide a new basis for evaluating the severity of CVDs, and provide new ideas for stratified treatment. Although a considerable number of studies have been carried out on the relationship between the metabolites of gut microbiota and CVDs, most of the current research is still in the basic experimental stage, and the specific mechanism has not been fully elucidated. Therefore, more high-quality, large sample size, clinical randomized control trials are needed to determine the safety and efficacy of targeting gut microbiota for the treatment of CVDs. Furthermore, more studies utilizing next-generation high-throughput sequencing technology and bioinformatics methods are needed to understand how gut microbiota interacts with surrounding organs and tissues, and to clarify their underlying molecular mechanisms in disease susceptibility.

Many new studies continue to emerge, providing intriguing reference for exploring the association between gut microbiota and CVDs. Based on previous studies, we collected evidence that some pathobionts, such as *Prevotella copri,* contributed to CVDs [269], while others, such as *A. muciniphila,* protected against CVDs [439]. The compositional pattern changes in gut microbiota, such as the *Firmicutes* to *Bacteroidetes* ratio, in different CVDs risks have been the hot points of gut microbiota research. Importantly, most clinical studies have suggested that the diversity, richness and evenness of gut microbiota are closely associated with CVDs [602, 603]. And some studies added bacteria to animals to observe the development of CVDs to explore the direct effect of gut microbiota on CVDs risk. However, in humans, it is unknown whether CVDs disrupt the balance of gut microbiota or whether the imbalanced gut microbiota can drive or initiate the occurrence of CVDs. We propose that these two processes would promote each other. Dysbiosis might also increase CVDs risk [413]. In turn, decreased heart function in CVDs disrupts the microenvironment of gut microbiota [62]. Furthermore, there is still a lack of research on how gut microbiota metabolites directly or indirectly affect disease susceptibility, on whether targeting this pathway can reduce the risk of diseases, and on the value of using gut microbiota metabolites to measure the risk, progression, and prognosis of disease. Of note, most studies did not explore the functional alterations and downstream consequences of dysbiosis. Thus, the detailed mechanisms of the direct participatory role of gut microbiota in CVDs need further investigation.

Moreover, some gut microbiota metabolites play a harmful role in the occurrence and development of CVDs. For example, TMAO directly or indirectly participates in the pathogenesis of CVDs [64]. The mechanism involves inhibiting cholesterol metabolism, inducing platelet aggregation and thrombus formation, and promoting atherosclerosis [64]. Besides, SCFAs play a key role in maintaining gut barrier function and have a positive effect on cardiovascular health. However, the impact of other metabolites, particularly BAs, on regulating the occurrence and development of CVDs is still controversial. Although some studies have shown that fasting plasma BAs inhibit atherosclerosis [369], some studies have also found that there is no statistically significant difference in fasting plasma BAs concentrations between normal-weight and obese people [604]. Notably, Chávez-Talavera et al. suggested that the proportion of different types of BAs, clinical-biological differences between the studied patient populations, and the heterogeneity of statistical analyses applied may explain the difference between studies [605]. From this, we speculate that the gut microbiota may play a role by affecting the ratio and composition of different types of gut microbiota-produced metabolites, and the specific relationship between gut microbiota-produced metabolites and CVDs needs to be further investigated.

In addition, the role of downstream signaling pathways regulated by gut microbiota-produced metabolites in the development of CVDs is not completely clear. Different studies reported inconsistent results. Although it is widely accepted that FXR and TGR5 mediate BAs metabolism [153], the effect of FXR and the TGR5-induced downstream signaling pathway on CVDs is still controversial in different studies [606, 607]. Some studies have suggested that the inhibition of FXR is beneficial for CVDs [219, 606]. For example, in vitro, upregulating the expression of FXR in cardiomyocytes induces cardiomyocyte apoptosis and reduces cardiomyocyte viability [606]. Deletion of the FXR receptor can significantly reduce the necrotic myocardial area, increase the cardiac ejection fraction, and improve cardiac structure and remodeling after myocardial infarction in mice [606]. However, another animal experiment has shown that the activation of FXR is beneficial [607]. Through activation of FXR, BAs can attenuate the development of atherosclerosis, improve lipid structure, and affect vascular tone [607]. Thus, further studies should explore the downstream pathways regulated by metabolites to identify targeted therapies for CVDs.

Another controversial topic is whether regulating these gut microbiota-produced metabolites has therapeutic effects on CVDs. Studies have also placed a spotlight on the treatment of CVDs by targeting gut microbiota and related metabolites. Diet regulation is the main application that impacts gut microbiota composition. Traditional dietary adjustments could benefit CVDs [120]. Although a high‐fiber diet or a diet supplemented with SCFAs had a protective effect on mice with hypertension and heart failure [407], the effect and mechanism of other diets such as BCAAs supplementation on CVDs still need more clinical and basic studies. Other promising treatments include probiotics and prebiotics and FMT treatment. Probiotics and prebiotics are supplementary approaches to manipulate the gut microbiome [608]. However, the effect of probiotics and prebiotics is only transient [609, 610], and has not been recommended by most clinical guidelines [611]. More importantly, the application of FMT is limited by potential adverse complications after bringing both beneficial and harmful bacteria [612, 613]. In summary, targeting gut microbiota and metabolites could be a promising therapy but more studies are needed before they can be used in clinical applications.

In the future, high-tech means involving bacterial gene sequencing should be used to detect the structure and function of gut microbiota metabolites, so as to formulate more effective targeted disease intervention programs, and provide new ideas for the improvement of health and the treatment of CVDs [614]. The emergence of a new generation of metagenomics and the development of bioinformatics will help us to further study the production and mechanism of microbial metabolites. A large number of high-quality studies are still needed to further confirm how the gut microbiota converts dietary and endogenous molecules into metabolites that communicate with the host’s peripheral organs and tissues, which will also facilitate the development of therapies targeting gut microbiota and point out new methods for the prevention and treatment of CVDs.

## Data Availability

Not applicable.

## Abbreviations

CVDs: Cardiovascular diseases
*A. **muciniphila*: *Akkermansia* *muciniphila*
LPS: Lipopolysaccharide
NOD: Nucleotide-binding oligomerization domain
NLRP: NOD-like Receptor Family, leucine-rich repeat, pyrin domain containing
HPA: Hypothalamic–pituitary–adrenal
RAS: Renin-angiotensin system
BAs: Bile acids
SCFAs: Short-chain fatty acids
TMAO: Trimethylamine N-oxide
BCAAs: Branched-chain amino acids
GPR: G protein-coupled receptor
Olfr78: Olfactory receptor 78
NF-κB: Nuclear factor kappa-B
BSH: Bile salt hydrolase
FMO3: Flavin monooxygenase 3
FXR: Farnesoid X receptor
TGR5: Takeda-G-protein receptor 5
mTORC1: Mammalian target of rapamycin complex 1
TGPR: G-protein-coupled receptors
GLP-1: Glucagon-like peptide 1
PYY: Peptide YY
mTORC1: Mammalian target of rapamycin complex 1
FOXA2: Forkhead box protein A2
FGF: Fibroblast growth factor
FXR: Farnesoid X receptor
TLRs: Toll like receptors
NLRs: Nod-like receptors
CAD: Coronary artery disease
MI: Myocardial infarction
T2D: Type 2 diabetes
LDLR: Low-density lipoprotein receptor
Lab4P: Lab4 probiotic consortium (*Bifidobacterium bifidum*, *Bifidobacterium animalis* subsp. *Lactis* and two strains of *lactobacillus acidophilus* plus *lactobacillus plantarum* cul66
BFM: *Bifidobacterium breve* Cect7263
DMB: 3,3-Dimethyl-1-butanol, an inhibitor of trimethylamine formation
FMT: Fecal microbiota transplantation
HDACs: Histone deacetylases
TNF: Tumor necrosis factor
FOXP3: Forkhead box protein P3
HDL: High-density lipoprotein
LDL: Low-density lipoprotein
CA: Cholic acid
CDCA: Chenodeoxycholic acid
DCA: Deoxycholic acid
LCA: Lithocholic acid
UDCA: Ursodeoxycholic acid
BSH: Bile salt hydrolase
CYP7A1: Cholesterol 7-alpha hydroxy-lase
TMA: Trimethylamine
PAGln: Phenylacetylglutamine
AMPK: Adenosine monophosphate activating protein
FIAF: Fasting-induced adipose factor
PPAR-γ: Peroxisome proliferator-activated receptor
12-OH: 12-Hydroxylated
TDCA: Taurodeoxycholic acid
UCP1: Uncoupling protein 1
mTOR: Mammalian target of rapamycin
JNK: C-Jun N-terminal kinases
IRS: Insulin receptor substrate
T1D: Type 1 diabetes
MAPK: Mitogen-activated protein kinase
Wnt: Wingless and int-1
ERK: Extracellular regulated protein kinases
CREB: CAMP response element binding protein
GSK: Glycogen synthase kinase
PKA: Protein kinase A
S6K1: S6 kinase beta-1
PGC-1α: Peroxisome proliferator-activated receptor-α coactivator
NADPH: Nicotinamide adenine dinucleotide phosphate
ROS: Reactive oxygen species
HFrEF: Heart failure with reduced ejection fraction
TMAVA: N,N,N-trimethyl-5-aminovaleric acid
TML: Trimethyllysine
TAC: Transverse aortic constriction
AF: Atrial fibrillation
TCA: Taurocholic acid
BMI: Body mass index
DASH: Dietary approaches to stop hypertension
FMD: Fasting-mimicking diet
BFM: *Bifidobacterium breve* CECT7263
LC40: *Lactobacillus fermentum* CECT5716
LVEF: Left ventricular ejection fraction
TCM: Traditional Chinese medicine
